# Effects of iron oxide nanoparticles as *T*_2_-MRI contrast agents on reproductive system in male mice

**DOI:** 10.1186/s12951-022-01291-2

**Published:** 2022-03-02

**Authors:** Heyu Yang, Hui Wang, Chenghao Wen, Shun Bai, Pengfei Wei, Bo Xu, Yunjun Xu, Chaozhao Liang, Yunjiao Zhang, Guilong Zhang, Huiqin Wen, Li Zhang

**Affiliations:** 1 Department of Urology, The First Affiliated Hospital of Anhui Medical University, Institute of Urology, Anhui Medical University and Anhui Province Key Laboratory of Genitourinary Diseases, Anhui Medical University, Hefei, 230022 China; 2 Reproductive and Genetic Hospital, Department of Radiology, Anhui Provincial Hospital, The First Affiliated Hospital of USTC, Division of Life Sciences and Medicine, University of Science and Technology of China, Hefei, 230001 China; 3 School of Pharmacy, The Key Laboratory of Prescription Effect and Clinical Evaluation of State Administration of Traditional Chinese Medicine of China, Binzhou Medical University, Yantai, 264003 China; 4 School of Medicine and Institutes for Life Sciences, South China University of Technology, Guangzhou, 510006 China; 5 Department of Blood Transfusion, The First Affiliated Hospital of Anhui Medical University, Hefei, 230022 China; 6 Center for Scientific Research of the First Affiliated Hospital of Anhui Medical University, Hefei, 230022 China; 7 Anhui Provincial Institute of Translational Medicine, Hefei, 230032 China

**Keywords:** Magnetic resonance imaging, Contrast agents, Iron oxide nanoparticles, Reversible toxicities, Reproductive system

## Abstract

Iron oxide nanoparticles (IONPs)-based contrast agents are widely used for *T*_2_-weighted magnetic resonance imaging (MRI) in clinical diagnosis, highlighting the necessity and importance to evaluate their potential systematic toxicities. Although a few previous studies have documented the toxicity concerns of IONPs to major organs, limited data are available on the potential reproductive toxicity caused by IONPs, especially when administrated via intravenous injection to mimic clinical use of MRI contrast agents. Our study aimed to determine whether exposure to IONPs would affect male reproductive system and cause other related health concerns in ICR mice. The mice were intravenously injected with different concentrations IONPs once followed by routine toxicity tests of major organs and a series of reproductive function-related analyses at different timepoints. As a result, most of the contrast agents were captured by reticuloendothelial system (RES) organs such as liver and spleen, while IONPs have not presented adverse effects on the normal function of these major organs. In contrast, although IONPs were not able to enter testis through the blood testicular barrier (BTB), and they have not obviously impaired the overall testicular function or altered the serum sex hormones levels, IONPs exposure could damage Sertoli cells in BTB especially at a relative high concentration. Moreover, IONPs administration led to a short-term reduction in the quantity and quality of sperms in a dose-dependent manner, which might be attributed to the increase of oxidative stress and apoptotic activity in epididymis. However, the semen parameters have gradually returned to the normal range within 14 days after the initial injection of IONPs. Collectively, these results demonstrated that IONPs could cause reversible damage to the reproductive system of male mice without affecting the main organs, providing new guidance for the clinical application of IONPs as *T*_2_*-*MRI contrast agents.

**Graphical Abstract:**

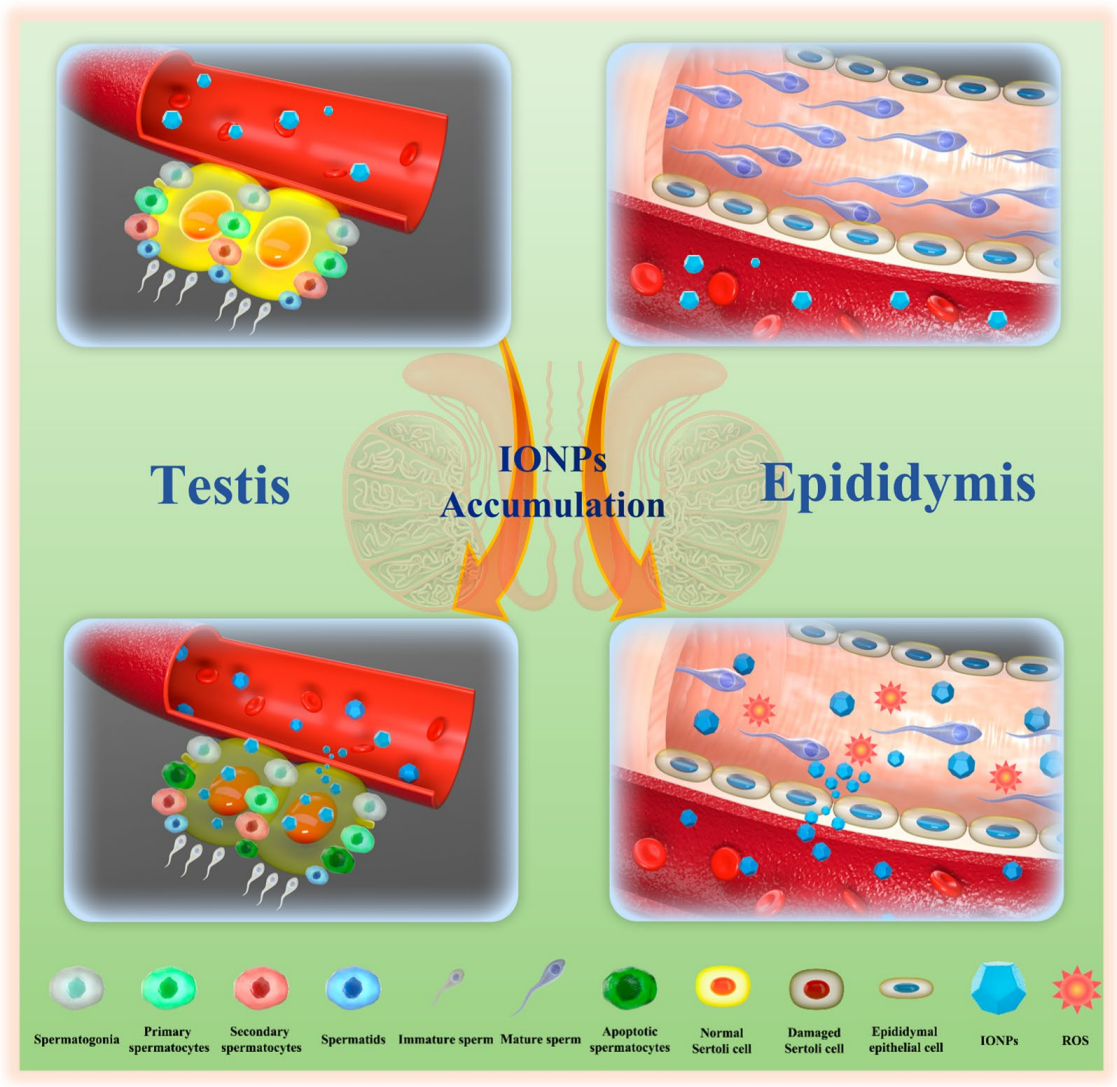

**Supplementary Information:**

The online version contains supplementary material available at 10.1186/s12951-022-01291-2.

## Introduction

Accurate and sensitive imaging examinations are of great importance to timely diagnosis and effective treatment of diseases. By possessing deep-tissue penetration, high spatial resolution with soft-tissue sensitivity, no radiation and several other unique advantages, magnetic resonance imaging (MRI) has been widely used in clinical practice [1, 2]. Compared with ultrasound, computerized tomography (CT), MRI plays an irreplaceable role in the diagnosis of cardiovascular diseases [3, 4], neurosurgical and neurological diseases [5, 6], arthritis and various types of tumors [7–9]. Although MRI has multiple scan sequences and parameters, such as *T*_1_/*T*_2_-weighted imaging (*T*_1_/*T*_2_-WI), diffusion-weighted imaging (DWI), dynamic contrast-enhanced imaging (DCEI) and magnetic resonance spectroscopic imaging (MRSI), it sometimes can not distinguish specific tumor tissues clearly due to the similar signal intensities of tumor tissues with adjacent normal tissues. Therefore, DCEI with intravenously-injected contrast agents to achieve signal difference amplification has gradually become a routine approach in clinical MRI diagnosis and prognosis evaluation [10, 11].

Superparamagnetic iron oxide nanoparticles (SPIONs) are currently the most successfully used nano-sized contrast agents for *T*_2_-WI and DCEI in MRI diagnosis [12, 13]. In spite of the growing variety of SPION formulations approved by the Food and Drug Administration (FDA) for use in humans as iron-deficiency therapeutics or as MRI contrast agents, e.g., Feridex^®^, Umirem^®^ and Resovist^®^ [14–16], constant reevaluation of the theranostic efficacy as well as the potential risk of side effects led to a changing area of their application in most cases. Therefore, the discovery of hidden side effects can bring enlightenment to clinical practice and guide the correct and reasonable use of contrast agents. On the basis of the present knowledge, these new-generation nanomaterial-based MRI contrast agents are primarily used for diagnostic applications in tumorous diseases, while they sometimes are also employed to diagnose benign diseases such as strain of joints. Accordingly, more close attention should be paid to their potential short- and long-term toxicities for avoiding harmful side effects in clinical diagnostics.

Nano-sized iron oxide contrast agents are generally regarded as biocompatible materials. Concretely, a parallel comparative in vivo mouse model study among extremely small iron oxide nanoparticles (ESIONs), manganese oxide nanoparticles (MnO NPs) and clinically used gadolinium-based contrast agents (GBCAs) has documented a better biosafety profile possessed by ESIONs than that of MnO NPs and GBCAs [17]. The follow-up study further revealed that compared with GBCAs, ~ 8 nm iron oxide nanoclusters exhibited a favorable biocompatibility and much lower risk of nephrogenic systemic fibrosis, suggesting the possibility of substituting GBCAs with iron oxide nanoclusters in clinical MRI diagnosis for those patients with impaired renal function [18]. Similarly, no significant toxicity concerns of dimercaptosuccinic acid (DMSA)-coated IONPs have been raised in a nonhuman primate model during the 120-day period, although DMSA-coated IONPs may show preferential accumulation in lung and brain tissues [19]. In addition to the intravenous injection of IONPs, the uptake, distribution and toxicity profile has also been evaluated in a rat model by intestinal perfusion of ultrasmall (4 nm core) iron oxide nanoparticles (USIONs) coated with tartaric/adipic acid. As a result, the in vivo absorption through the small intestine reached above 79% of the initial perfusion of USIONs, however, the intact particles around the intestinal microvilli have not caused significant tissue damage. Moreover, such high absorption levels have not compromised cell viability, demonstrated by cytotoxicity, reactive oxygen species (ROS) production, genotoxicity and lipid peroxidation assays in Caco-2 and HT-29 enterocytes-like models [20].

Notably, as male reproductive system has been regarded as vulnerable and even more sensitive to exogenous materials than other organ systems, in addition to the aforementioned systematic assessment of the functional damage of major organs like heart, liver, spleen, lung, kidney and hematopoietic system, potential side effects on male reproductive system had also been evaluated for a few types of materials. For one thing, some nanomaterials could pose an unfavorable effect on the male reproductive system via altering spermatogenesis and causing histopathological damage [21]. Among them, PbSe NPs were able to accumulate in the testis, followed by decrease in testis and epididymis coefficients, disorder of sex hormones, destruction of normal seminiferous tubule structures, reduction in both quantity and quality of sperms, further leading to an impaired fertility [22]. Similarly, repeated intravenous injections of Mn_3_O_4_ nanoparticles into male mice could cause particles accumulation in the testis, then triggered oxidative stress reflected by malondialdehyde (MDA) upregulation, disturbed the balance among sex hormones and finally displayed decreased fertility [23]. In addition, Sundarraj et al. have comprehensively demonstrated that when Fe_2_O_3_ NPs were intraperitoneally (i.p.) administered into male mice once a week for 4 weeks, Fe_2_O_3_ NPs would cross the BTB to accumulate in the testis, further leading to the histopathological lesions like vacuolization, detachment, and sloughing of germ cells, they have also revealed that oxidative stress and apoptosis were the main causes of testicular toxicity [24]. However, for another, several nanoparticles are free of harmfulness to the male reproductive system. For instance, different modifications of gold nanoparticles could accumulate in the testis of male mice, and even pass through the BTB to enter germ cells, while they have not presented any obviously adverse effect on normal fertility [25].

Intriguingly, nanomaterials with same component may have distinct or even opposite effects on male reproductive system under different conditions. For instance, many studies have reported the histological damage in testicular and epididymal tissues after exposure to zinc oxide nanoparticles (ZnO NPs) in vivo, including multinucleated giant cells formation, germ cell layers disorganization and immature germ cells detachment [26, 27]. However, given the biological activities in activating steroidogenic enzymes and alleviating oxidative stress, ZnO NPs supplementation could also minimize the adverse effects on the sperm parameters, histological structure of the testis and epididymis, which were commonly observed after nicotine exposure in male rats [28]. In other words, nanomaterials may have a negative or positive effect on male reproductive system, and it is not possible to deduce their potential reproductive toxicity.

With the widespread use of contrast agents in clinical practice, a considerable number of male patients within reproductive ages have necessarily received MRI examinations with intravenously injected contrast agents. Therefore, for these populations, the risk assessment of the reproductive function should be carefully conducted. Especially, the concrete profile of the reproductive system exposed to intravenously injected IONPs, such as the distribution of IONPs in reproductive organs, their ability to pass through reproduction-relevant physiological barriers and the effects on sperm parameters or sex hormones, are still largely unknown.

**Fig. 1:**
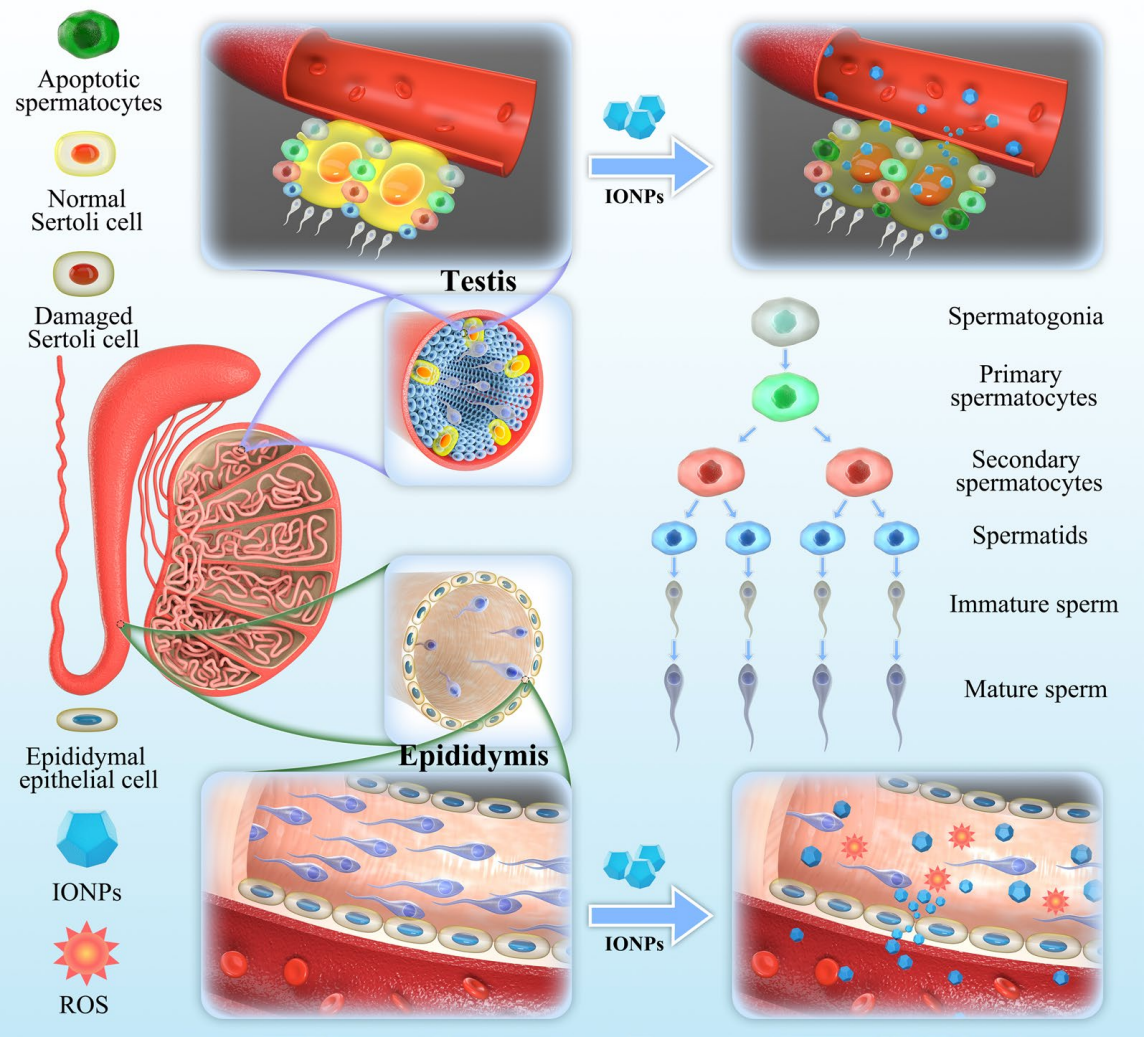
Schematic illustration of effects of intravenously-injected IONPs on testes and epididymides in male ICR mice

In this study, we have successfully designed the poly (acrylic acid) (PAA)-capped IONPs, and investigated their biodistribution, major organs toxicities, especially reproductive toxicities, which provided an important reference for use of IONPs as *T*_2_-MRI contrast agents. To mimic the clinical applications in terms of dosage, duration of treatment and route of administration, we intravenously injected different concentrations of IONPs via the tail vein into adult male ICR mice. Our results showed that IONPs had negligible toxicity in major organs such as heart, liver, spleen, lung and kidney, which was consistent with previous reports [14–19]. However, we found the accumulation of IONPs in epididymis, and IONPs were toxic to the reproductive system including testis and epididymis during short-term, resulting in impaired sperm quantity/quality and even apoptotic sperms (Fig. 1). Nevertheless, the sperm count and quality returned to the normal range after 2 weeks since initial injection of IONPs. These findings will provide a valuable enlightenment that the use of IONPs may lead to temporary but reversible impaired fertility, suggesting male patients who received MRI examinations with IONPs as *T*_2_-MRI contrast agents may need to delay fertilized behavior during a certain period.

## Materials and methods

### Materials

FeCl_3_·6H_2_O (98%), oleic acid (OA, 90%), 1-octadecene (ODE, 90%) and poly (acrylic acid) (PAA, Mw = 1800) were purchased from Sigma Chemical Ltd; sodium oleate (95%) was purchased from TCI Company; normal hexane, chloroform, ethanol, dimethyl sulfoxide were purchased from the Shanghai Reagent Company (Shanghai, China). All chemicals were analytical and used as received without further purification. Alanine aminotransferase (ALT, C009-2-1), aspartate aminotransferase (AST, C010-2-1), creatinine (Cr, C011-2-1) and blood urea nitrogen (BUN) (C013-2-1) assay kits were obtained from Jiangcheng Bioengineering Institute (Nanjing, China). Alpha-glucosidase activity assay kit (Colorimetric, ab174093) was purchased from Abcam (Cambridge, UK). CDC2 antibodies (DF6024) were purchased from Affinity Biosciences (Jiangsu, China). Cyclin B1 antibodies (55004-1-AP) and β-actin antibodies (20536-1-AP) were purchased from Proteintech Group, Inc. (Wuhan, China). HRP-conjugated anti-Rabbit IgG (E-AB-1003) were obtained from Elabscience Biotechnology (Wuhan, China). Cleaved caspase-3 antibodies (AC033), enhanced chemiluminescence (ECL) kit (P0018FS), H&E staining kit (C0105M) and TUNEL apoptosis assay kit (C1098) were purchased from Beyotime Biotechnology (Shanghai, China). The 0.45 μm nitrocellulose (NC) membrane (10-6000-03) was obtained from Cytiva (Shanghai, China). Immunohistochemical staining kit (SP-9000) and 3,3′-diaminobenzidine (DAB, ZLI-9018) were purchased from Zhongshan Golden Bridge Biotechnology (Beijing, China). Follicle-stimulating hormone (FSH, JYM0417Mo), luteinizing hormone (LH, JYM0341Mo) and testosterone (T, JYM0373Mo) ELISA kits were purchased from Wuhan jiyinmei Biotechnology Co., Ltd (Wuhan, China). Automatic Sperm Analyzer (ML-MD06200B) and Sperm Counting Chamber (ML-CASA60-4) were purchased from Nanning Song Jing Tianlun Biotechnology Co., Ltd (Nanning, China).

### Synthesis of iron oxide nanoparticles and surface modification

Monodisperse ultrasmall iron oxide nanoparticles were synthesized via a modified protocol [29]. The as-prepared iron oxide nanoparticles were redissolved into chloroform to achieve a stock solution (5 mg (Fe) /mL). 2 mL stock solution was mixed with PAA in 200 mL dimethyl sulfoxide (DMSO) by sonication. The molar ratio of the exchange ligand to Fe atoms was set roughly at 5 to 1 [30]. After 48 h, the hydrophilic PAA-capped iron oxide nanoparticles were collected by centrifugation. Then, the water-soluble nanoparticles were washed by deionized water, followed by redispersion into water.

### MR performance investigation and MR phantom analysis

MR investigation was carried out on Siemens 3.0 Tesla MR scanner. *T*_2_ weighted MR images were acquired using Turbo Spin Echo (TSE) sequence with a knee coil. Imaging parameters for *T*_2_ MR imaging were as follows: repetition time (TR): 5000 ms, echo time (TE): 40, 103, 206 ms, FOV: 240 × 240 mm.

### Characterization

The phase of the as-prepared product was characterized by X-ray power diffraction (XRD) analyses; which was carried out on a Philips X’Pert PRO SUPER X-ray diffractometer equipped with graphite monochromatized Cu Ka radiation and the operation voltage and current were maintained at 40 kV and 40 mA; respectively. Transmission electron microscopy (TEM) was investigated by Hitachi 7700. Energy dispersive X-ray spectroscopy (EDS) was performed with a scanning electron microscopy (Carl Zeiss Supra 40, Germany) equipped with EDAX microanalysis. XPS was recorded by a photoelectron spectrometer (ESCALAB, 250Xi, Thermo Fisher, USA). Thermogravimetric analysis (TGA) was performed on a TGA thermal analyser (SDT Q600, TA Instruments, USA). The magnetic properties of the samples were measured by a superconducting quantum interface device (SQUID) magnetometer (MPMS XL, Quantum Design, USA). Particle size and zeta potential were examined by Malvern Zeta analyzer (ZS-90 nanosizer, Malvern Instrument, UK). FT-IR spectra were measured using a FT-IR spectrometer (Nicolet 8700, Thermo Scientific, USA). All the concentrations of our products were measured by inductive coupled plasma atomic emission spectrometer (ICP-AES) (iCAP 7400, Thermo Fisher, USA).

### Animal models

The male ICR mice (5–6 weeks old, approximately 20 g body weight/BW) were purchased from the Animal Experiment Center of Nanjing University (Nanjing, China), and housed in an isolated animal room with water and rodent food supplements. The animals were acclimated to the environment for 1 week prior to the experiments. All animal experimental protocols and procedures were reviewed and approved by the Institutional Animal Care and Use Committees on Animal Care of Anhui Medical University (Approval No. LLSC20211064).

### In vivo biocompatibility assay

For in vivo biocompatibility evaluation of IONPs, ICR mice were injected with different concentrations of IONPs solution via tail vein (100 μL, 2.5 mg/kg BW, 10 mg/kg BW and 20 mg/kg BW, BW will be omitted for conciseness hereafter), and PBS was injected as a control. At predetermined time points, the mice were sacrificed, and the organs were harvested, including the brain, heart, liver, spleen, lung, kidney, testis and epididymis. The amounts of Fe ions in various organs were quantitatively determined by ICP-AES.

### In vivo safety evaluation

To investigate the toxic profiles of IONPs, the ICR mice were divided into four groups that were intravenously administered PBS and IONPs at dosages of 2.5, 10 and 20 mg/kg, respectively. Body weights, water intake and food consumption of each mouse were measured at predetermined intervals throughout the course of the study after intravenous administration until day 28. After 1, 3, 7, 14 and 28 days, the mice were sacrificed, and the organs were harvested for H&E staining. To further evaluate the safety in vivo, the serum levels of urea nitrogen (BUN), serum creatinine (CRE), alanine aminotransferase (ALT) and aspartate transaminase (AST) were analyzed using commercial kits.

### Sperm parameters analysis

To evaluate the toxicity of IONPs to sperms, the ICR mice were intravenously administered PBS and IONPs at dosages of 2.5, 10, and 20 mg/kg, respectively. These mice were closely monitored for 1, 3, 7, 14 and 28 days after the initial drug exposure. Then, the left cauda epididymis was removed and immediately put into 50 mL of preheated DMEM medium at 37 °C. Afterwards, it was cut into small pieces and incubated at a constant temperature of 37 °C for 30 min to let the sperms release to the medium sufficiently. The sperms suspension was analyzed by a computer-aided semen analysis system (ML-MD06200B, Nanning Song Jing Tianlun Bio-technology Co., Ltd.) for the sperm comprehensive evaluation, including the concentration, mobility, vitality, and sperm motion parameters.

### In vivo toxicity evaluation of testis

To investigate the toxicity of IONPs to testis, the ICR mice were intravenously administered PBS and IONPs at dosages of 2.5, 10 and 20 mg/kg, respectively. After the initial drug exposure at 1, 3, 7, 14 and 28 days, the mice were sacrificed, and the testis was harvested. The testis image was recorded and testis index (testis weight/body weight, w/w %) was calculated after intravenous administration. Then, the H&E staining was used to evaluated the damage of testis caused by IONPs, and the amounts of Fe ions in testis were quantitatively determined by ICP. In addition, the serum of mice was collected to measure the sex hormones including follicle-stimulating hormone (FSH), testosterone (T) and luteinizing hormone (LH) by ELISA.

### In vivo toxicity evaluation of epididymis

To know the toxicity of IONPs to epididymis, the ICR mice were intravenously administered with PBS and IONPs at dosages of 2.5, 10, and 20 mg/kg, respectively. After the initial drug exposure at 1, 3, 7, 14 and 28 days, the mice were sacrificed, and the epididymis was harvested. The epididymis index (epididymis weight/body weight, w/w %) was calculated after intravenous administration. Then the amounts of Fe ions in epididymis were quantitatively determined by ICP-AES, and the H&E staining and TUNEL apoptosis assay were used to evaluated the damage of epididymis caused by IONPs. Images were captured with a fluorescence microscope (Olympus BX61W1with Fluoview FV1000 software, Japan), and then analyzed using the ImageJ software. In addition, the right epididymis tissue homogenates were prepared to measure the epididymal MDA and T-SOD by following the commercial kit’s protocol.

### Western blotting

Briefly, the testicular tissues were homogenized and lysed in ice-cold RIPA buffer containing protease inhibitors, the homogenates were then centrifuged at 13,000 g for 10 min at 4 °C, and the supernatants were stored at -80 °C until analyzed. Proteins were separated by 12% SDS-PAGE and transferred to NC membrane. After blocking with 5% nonfat milk dissolved in TBST, NC membrane was incubated at 4 °C with specific primary antibodies at the proper dilutions (1:1000 for CDC2/Cyclin B1, 1:2000 for β-actin) overnight, after extensive TBST wash followed by secondary antibodies (1:5000) incubation for 2 h at room temperature and extensive TBST wash, the protein signals were visualized by ECL with a CLINX ChemiScope 5600 instrument (Shanghai, China). The relative protein levels were analyzed and quantified relative to β-actin by ImageJ software (NIH, USA).

### Evaluation of epididymal function

The α-glucosidase (α-GC) activity assay was conducted according to the manufacturer’s protocols. Briefly, the epididymis was weighted and homogenized in 1 mL ice-cold α-GC assay buffer, and then the homogenates were centrifuged at 13,000×g for 10 min at 4 °C to obtain the supernatant. 50 µL supernatant of samples, 47 µL α-GC assay buffer and 3 µL α-GC substrate were mixed and added to each well. The OD values were immediately measured at 410 nm wavelength in a kinetic mode for 60 min with 5 min interval. The activity (mU/g tissue) of α-GC in each sample was calculated as follows: 20 × (Sa/Ss) / epididymis weight. Sa: slope of the enzyme activity (OD/nmol); Ss: slope of the standard curve (OD/min).

### Immunohistochemical (IHC) staining

Briefly, epididymal tissues were fixed in 10% formalin for 48 h, embedded in paraffin, and cut into 5 μm-thick sections. Xylene and ethanol at a series of concentrations (100%, 95%, 75%) were applied to deparaffinization and dehydration, respectively. Then, the sections were placed in boiling solution (0.01 M citric acid buffer at pH 6.0) for 15 min to complete antigen retrieval. To block the endogenous peroxidase activity, 3% hydrogen peroxide solution was used to incubate the sections for 10 min at room temperature. After wash with PBS, the sections were blocked with 10% goat serum albumin followed by incubation with cleaved caspase-3 antibodies (1:100 dilution) at 4 °C overnight. After wash with PBS for three times, biotinylated goat anti-rabbit IgG (1:200 dilution) and HRP-labelled streptavidin were added to incubate with the sections at 37 °C for 30 min, respectively, with extensive wash between each procedure. Finally, the immunoreactivity was visualized with DAB.

### Statistical analysis

All results were expressed as the means ± standard error of mean (S.E.M.) as indicated. Student’s t test was used for the comparison of differences between two groups, while one-way analysis of variance (ANOVA) was applied to evaluate the statistically significant differences among multiple groups. All statistical analyses were performed using SPSS 19.0 (SPSS Inc., Chicago, IL, USA). The threshold for statistical significance was P < 0.05.

## Results and discussion

### Preparation of hydrophilic iron oxide nanoparticles

**Fig. 2:**
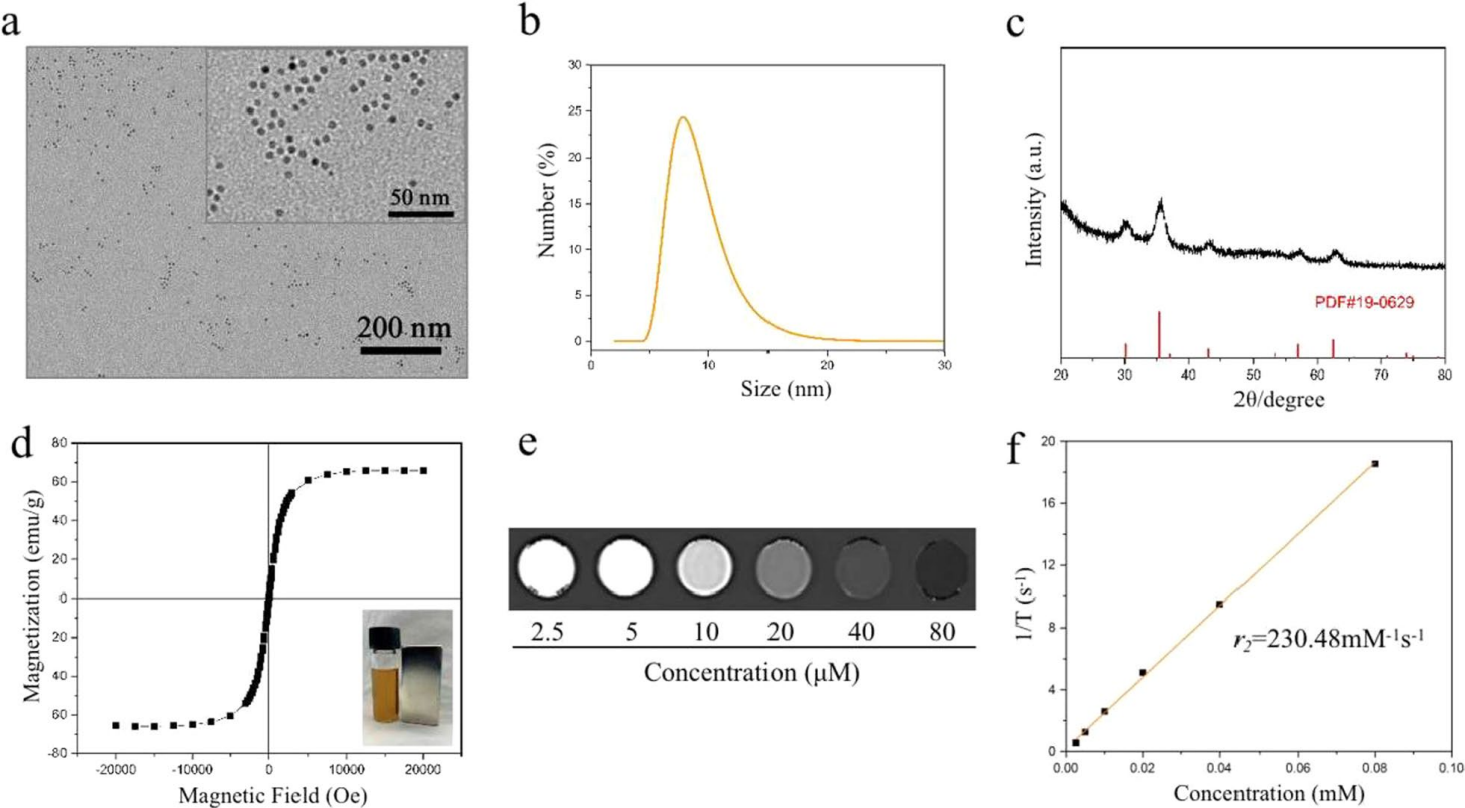
Characterization of IONPs. **a** TEM image and **b** size distribution measured by DLS of IONPs dispersed in aqueous solution. **c** XRD patterns of IONPs. **d** The M-H loop of IONPs at 300 K. Inset showed the response of hydrophilic IONPs to a magnet. **e** *T*_2_-weighted image and **f** transverse relaxivity (*r*_*2*_) of IONPs at 3.0 T (R^2^ = 0.999)

It is well-established that surface modifications of IONPs could not only effectively enhance the stability of nanoparticles but also potentially affect the biocompatibility and pharmacokinetics of nanoparticles in vivo [31]. Some studies have found that positively-charged IONPs possessed a higher affinity to attach to the cell membranes, and they were more likely to be internalized in much larger amounts compared with negatively-charged IONPs [32]. Moreover, greater bioaccumulation, more protein adsorption and significant toxicity were also observed in positively-charged IONPs [32, 33]. Hypersensitivity reaction reported in most iron-based agents has recently attracted close attention [34, 35]. Attractively, lower probability of hypersensitivity was benefited from the high carboxyl group coating density of negatively-charged IONPs [36]. According to the aforementioned discoveries, a kind of low molecular weight poly (acrylic acid) (PAA) was selected to decorate and manufacture the monodisperse ultrasmall iron oxide nanoparticles via a modified protocol. The as-prepared 6 nm-diameter oleic acid-capped iron oxide nanocrystals initially dispersed in chloroform (Additional file 1: Fig. S1) [29]. After ligand exchange on nanoparticle surfaces by PAA, the hydrophilic PAA-capped iron oxide nanoparticles (IONPs) were obtained [30]. IONPs dispersed into aqueous solution were then examined by transmission electron microscopy (TEM) and dynamic light scattering (DLS) to demonstrate uniformity of size and distribution. TEM revealed the particle size of IONPs was ~ 6 nm (Fig. 2a), and the hydrodynamic diameter in aqueous solution measured by DLS was 8.62 ± 2.18 nm (Fig. 2b), validating its excellent monodispersion and stability. The overall zeta potential of IONPs (-22 mV) was measured using zeta seizer, indicating successful surface functionalization of the as-prepared hydrophilic nanoparticles. The FT-IR spectra further confirmed the surface modification of IONPs by PAA (Additional file 1: Fig. S2). The adsorption bands at 2923 and 2853 cm^−1^ were attributed to the -CH_2_- groups [37]. The peak at 1718 cm^−1^ was assigned to the carbonyl group (C = O) [30]. The appearance of two peaks at 3440 and 1631 cm^–1^ were ascribed to the -OH bending vibration, which was consistent with previously reported PAA-coated iron oxide nanoparticles [30, 37, 38]. The X-ray diffraction (XRD) pattern of IONPs was illustrated in Fig. 2c, which corresponded to magnetite (JCPDS 19–0629). IONPs dispersed in aqueous solution could form a stable suspension, and it showed a good response to the magnet (inset of Fig. 2d). The energy dispersive X-ray spectroscopy (EDS) spectrum of IONPs was shown in Additional file 1: Fig. S3, and the incorporated Fe, O, and C elements had an atom ratio of 1.00:2.21:13.21. In addition, XPS was used to study the surface element component of samples. The Fe/O/C atom ratio from the high- resolution XPS scans was determined to be 0.29:6.62:93.08. As shown in Additional file 1: Fig. S4, two peaks at 711 eV and 725 eV were assignable to the characteristic peaks of Fe 2p_3/2_ and Fe 2p_1/2_ for IONPs [39]. We then used thermogravimetric analysis (TGA) to measure the content of surface functionalized decorations. The loss between 300 and 500 °C was mainly attributed to the weight loss of surface ligands. The TGA result showed that the weight ratio of surface ligands was approximately 65% (Additional file 1: Fig. S5). A SQUID test was carried out to investigate the magnetic property of the acquired IONPs. As shown in Fig. 2d, IONPs had a saturation magnetization (Ms) of 65.95 emu/g Fe at room temperature.

### Relaxometric property of IONPs

To ascertain the MRI relaxation property of IONPs, the transverse relaxation time of IONPs with different Fe concentrations was measured by a clinical 3.0 T MRI scanner. An enhancement in the *T*_2_-weighted MR signals was observed as the concentration of IONPs increased (from 0.0025 to 0.08 mM of Fe), leading to a raise in the signal of the corresponding MR images (Fig. 2e). The corresponding transverse relaxivity (*r*_2_) of IONPs was calculated to be 230.48 mM^−1^ s^−1^, indicating a good MR imaging behavior (Fig. 2f).

### Investigation of the biocompatibility of IONPs in vivo

**Fig. 3:**
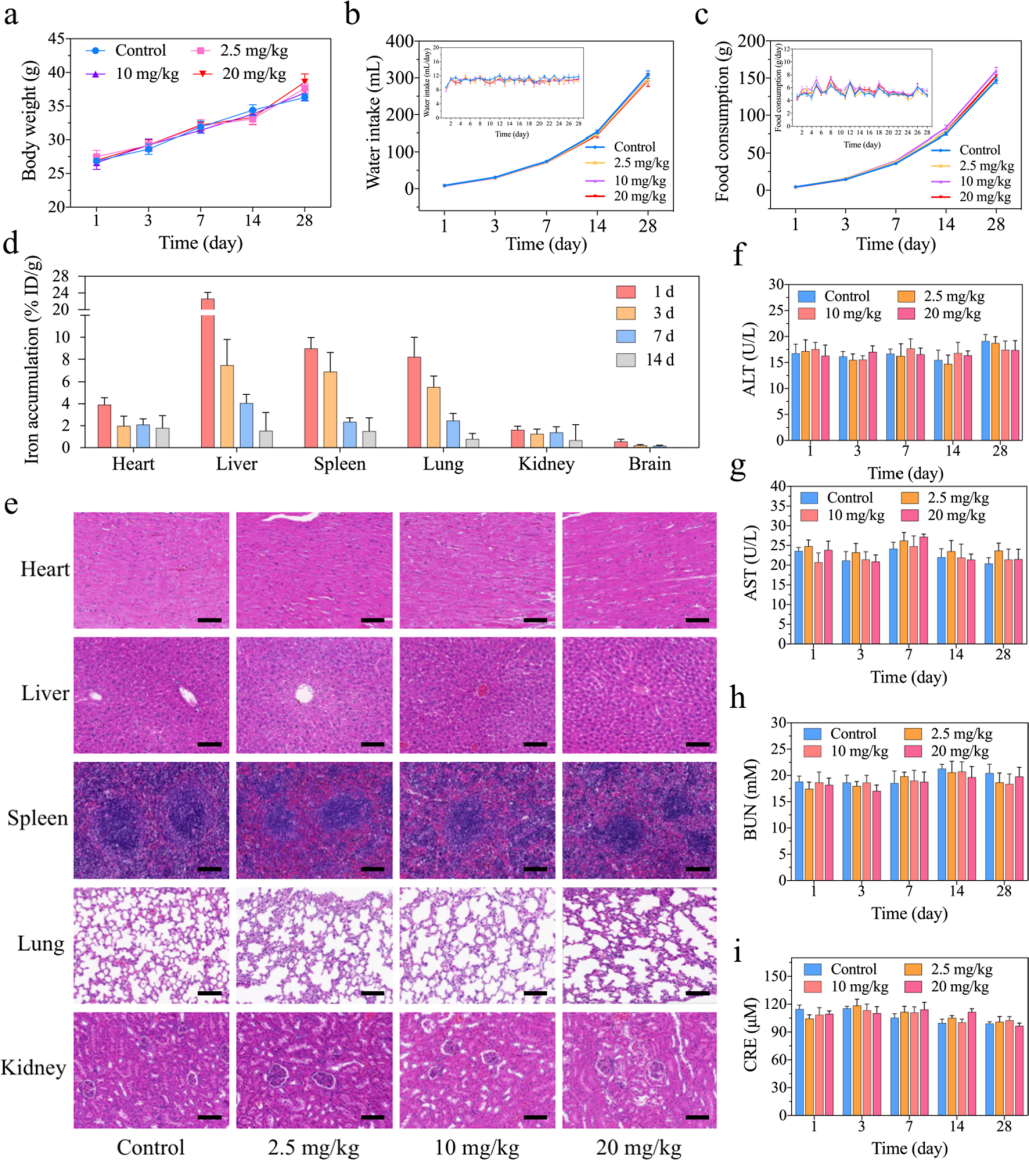
The biocompatibility and distribution of IONPs for major organs in vivo. **a** Body weight curves after intravenous injection of IONPs. **b**, **c** Cumulative and daily water intake (**b**) or food consumption (**c**) after intravenous injection of IONPs. **d** The distribution of Fe ions in the major organs of ICR mice at 1, 3, 7 and 14 days after initial IONPs exposure. **e** Histopathological examination of the major organs of ICR mice after intravenous injection of IONPs at various concentrations for 1 day. Scale bar, 100 μm. **f**, **g** Alanine aminotransferase (ALT) (**f**) and aspartate aminotransferase (AST) (**g**) values of ICR mice for assessing liver function after intravenous injection of IONPs at various concentrations for 1 to 4 weeks. **h**, **i** The parameters of renal function including blood urine nitrogen (BUN) (**h**) and creatinine (CRE) (**i**) of ICR mice after intravenous injection of IONPs with three concentrations for 1 to 4 weeks. Data were expressed as the Mean ± S.E.M., n = 5

Intravenous injection is the most routinely used approach for administration of IONPs as MRI contrast agents, and intravenously injected IONPs could also circulate through the bloodstream to multiple organs. In general, IONPs are selectively taken up by the liver and spleen [17, 18, 40], while a few reports also documented the retention or elimination of IONPs in the lung or kidney [41, 42], subsequently raising the toxic concerns in major organs. As IONPs were injected intravenously, the hemolysis rate of IONPs was firstly evaluated, and the rate was calculated to be almost zero, indicating the excellent haemocompatibility of IONPs and promising intravenous administration approach (Additional file 1: Fig. S6). Secondly, the potential toxicity of IONPs to major organs has been evaluated. Body weight (BW) is one of the simple, intuitive and effective indexes to reflect systemic toxicity, as shown in Fig. 3a, compared with the PBS group, there was no BW loss in the IONPs group treated with different doses. The BW of mice injected with high dose of IONPs (26.97 ± 0.34 g) on the first day were equivalent to the control group (26.87 ± 0.43 g), and on the 28th day, the BW of mice administrated with high-dose IONPs (38.57 ± 1.22 g) were even slightly heavier than the control group (36.29 ± 0.54 g), which exhibited their negligible systemic toxicity in vivo. It is well-established that water intake and food consumption of experimental animals are also classical indexes to reveal the possible systematic toxicity [24, 43, 44]. Thus, we have continuously monitored water intake and food consumption of mice after low-, middle- and high-dose IONPs administration. As a result, there were no significant changes of water intake and food consumption in the IONPs group treated with different doses, compared with that of each control group from 1 to 28 days post-injection (Fig. 3b, c). To lend more direct evidence regarding IONPs on specific major organs, the accumulation profile of IONPs in heart, liver, spleen, lung, kidney and brain was depicted by inductively coupled plasma atomic emission spectrometer (ICP-AES) on 1, 3, 7 and 14 days after intravenous injection of IONPs. As a result, the average amounts of Fe ions retained in organs were 22.63% ID/g (liver), 8.98% ID/g (spleen), 8.22% ID/g (lung), 3.92% ID/g (heart), 1.63% ID/g (kidney) and 0.56% ID/g (brain) in descending order 24 h after initial injection (Fig. 3d). Obviously, IONPs mainly accumulated in liver, spleen and lung, while negligible amounts were distributed in brain, which may be hampered by blood–brain barrier (BBB). With the increase of time, the concentration of Fe ions in the tissues decreased rapidly. For example, the amount of Fe ions in liver reduced from 22.63% ID/g of the first day to 4.07% ID/g of the seventh day, and the decrease trend was also observed in the other organs. Later on, the histopathological examination (hematoxylin–eosin, H&E) staining was utilized to support the biosafety profile of IONPs in major organs (Fig. 3e and Additional file 1: Figs. S7–S10), in which no abnormal changes in the pathological cellular structures or hyperemia, edema, cell death and other obvious injuries were observed, indicating no significant systematic risk of the IONPs to ICR mice following intravenous injection. In details, normal lung parenchyma was observed, and the alveoli presented a vacuolated thin-walled structure. In the hepatic tissues, hepatocytes arranged in radial lines around the central vein. Meantime, the red pulp and follicles of the white pulp were well organized, indicating physicological spleen tissues. For the kidney, the mesangium appeared normal without obvious damage and the glomerular capillary or Bowman's capsule was observed in a typical nephron. Lastly, the myocardium structure was complete and myocardial cell morphology was normal. In addition, the most important serum biochemical parameters, including alanine aminotransferase (ALT), aspartate aminotransferase (AST), blood urine nitrogen (BUN), and creatinine (CRE), were used to present the function of main organs after IONPs administration (Fig. 3f–i). The results showed that these biochemical parameters in IONPs-treated mice with doses from 2.5 mg/kg to 20 mg/kg at each timepoints (up to the 28th day) were within the normal range, in other words, they have not significantly altered compared with the control group. In addition, there were no significant differences among each groups treated with low-, middle- and high-dose IONPs, respectively. For instance, no obvious increase in ALT value was documented on the first day from low-dose group (17.18 ± 2.17 U/L) to middle-dose group (17.58 ± 1.31 U/L) or high-dose group (16.29 ± 2.07 U/L). Collectively, all the above results indicated that there were no obvious toxicities to major organs of IONPs, which was in consistent with the previous reports regarding systematic toxicity evaluation of IONPs [6–8].

### Evaluation of the sperm quantity and quality in vivo

**Fig. 4:**
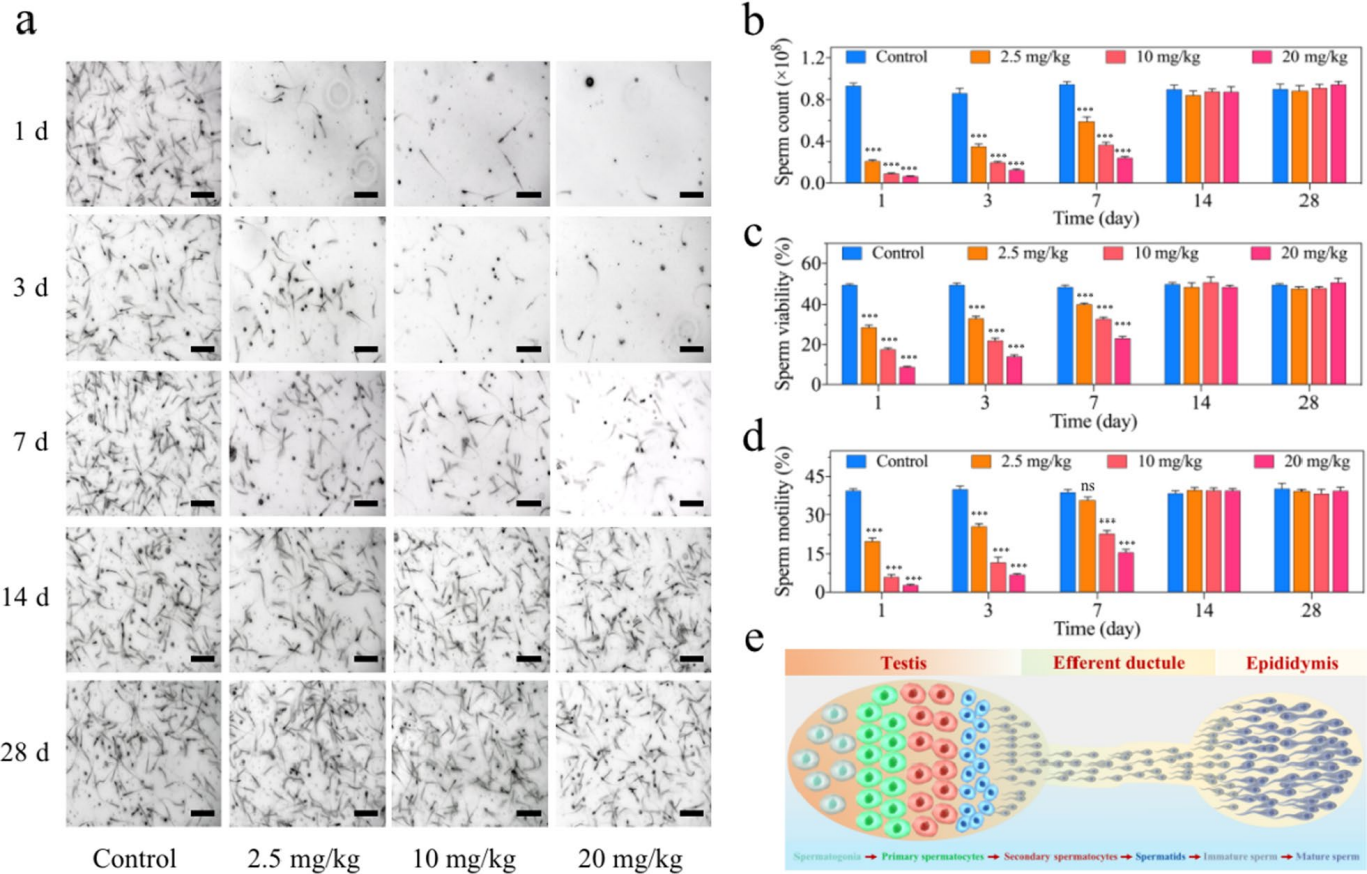
The semen analyses for male ICR mice after IONPs injection. **a** The photos of sperms suspension of ICR mice under microscope at 1, 3, 7, 14 and 28 days after initial IONPs exposure. Scale bar, 100 μm. **b–d** The sperm count (**b**), motility (**c**) and viability (**d**) analyses of ICR mice at 1, 3, 7, 14 and 28 days after intravenous injection of IONPs. Data were expressed as the Mean ± S.E.M., n = 5, ns, not significant, ***P < 0.001. **e** Schematic illustration of sperm production, transport and storage

Reproductive diseases have raised growing concerns worldwide and the male factor accounts for a considerable proportion of the problems. Sperm cell is responsible for carrying the paternal genetic complement to the oocyte and forming an euploid zygote, which holds great importance to maintain normal fertility [45]. However, compared with somatic cells, spermatozoa are relatively vulnerable to oxidative stress, external stimuli and many other factors, owing to their limited capability of antioxidant protection and DNA repair mechanisms [46]. Therefore, the impact of IONPs on male reproductive system needs to be carefully evaluated, in addition to the systematic toxicity assessment of the aforementioned major organs. Accordingly, the semen analysis profiles on male ICR mice intravenously (i.v.) injected with IONPs at low-, middle- or high-dose (2.5, 10, and 20 mg/kg, respectively) were closely monitored for 28 days. As a result, the amount of normal sperms in the epididymis significantly reduced in a dose-dependent manner. On the first day after injection, the number of sperms dropped precipitously, we could only observe the presence of a small amount of sperms (approximately 21.7% of the normal value) in low-dose group and even hardly counted the viable sperms (less than 8% of the normal value) in high-dose group (Fig. 4a). This dose-dependent reduction of sperm quantities also appeared on the 3rd and 7th days after initial injection, although the sperm counts have gradually increased, as compared with the first day post-injection. For instance, the sperm counts in epididymal tissue suspensions were approximately 7.1%, 14.4% and 25.6% of the normal value on the first, third, and seventh day after high-dose injection, respectively (Fig. 4b). Notably, the sperm quantities have returned to the physiological range on the 14th day, and kept normal until the observed duration of 28 days (Fig. 4a, b). As for male reproductive function, sperm quality possesses equal or even more importance than sperm quantity [47]. Thus, sperm qualities including viability and motility were evaluated following IONPs injection. Consequently, in consistent with the alternation in sperm quantities, IONPs impaired sperm qualities in a dose-dependent manner at each checkpoints of the first, third, seventh day post-treatment, and the harmful effect achieved the peak on the first day after low-, middle- or high-dose injection. Concretely, on the first day after injection of IONPs, the sperm viability rate of the low-dose group (28.56 ± 1.14%) was significantly lower than that of the control group (49.79 ± 0.57%), let alone the middle-dose group (17.56 ± 0.81%) or the high-dose group (8.75 ± 0.32%) (Fig. 4c). At the meantime, sperm motility showed almost identical changing pattern with sperm viability, no matter which doses of IONPs were administrated in each timepoints. For instance, the motility rate was extremely lower in the high-dose group (2.74 ± 0.38%), compared to that in the control normal group (39.43 ± 0.77%) (Fig. 4d). Notably, in accordance with that the sperm counts gradually increased and returned to the normal range on the 14th day, the sperm viability and motility also recovered to the normal range in the same duration, suggesting that the toxicities of IONPs to sperms quantity and quality were temporary and could be reversible within 2 weeks. In consideration that the sperms were released from the epididymal fragment to the buffer followed by measurement [48], sperms with normal viability and motility in the control group could almost entirely release from the epididymis into the buffer, while in IONPs-injected groups, the total counted number of sperms might be lower than they actually were, demonstrating the proportion of functional sperms should be further lower than currently estimated. Collectively, these findings raised an important clinical implication that there would be a temporary but recoverable loss of sperm quantity and quality after intravenous injection of IONPs. If a male patient has received MRI examination with IONPs as contrast agents, the fertilized behavior should be delayed until the semen parameters are recovered, which may need some duration.

### Evaluation of hormones and testicular indexes after IONPs administration

**Fig. 5:**
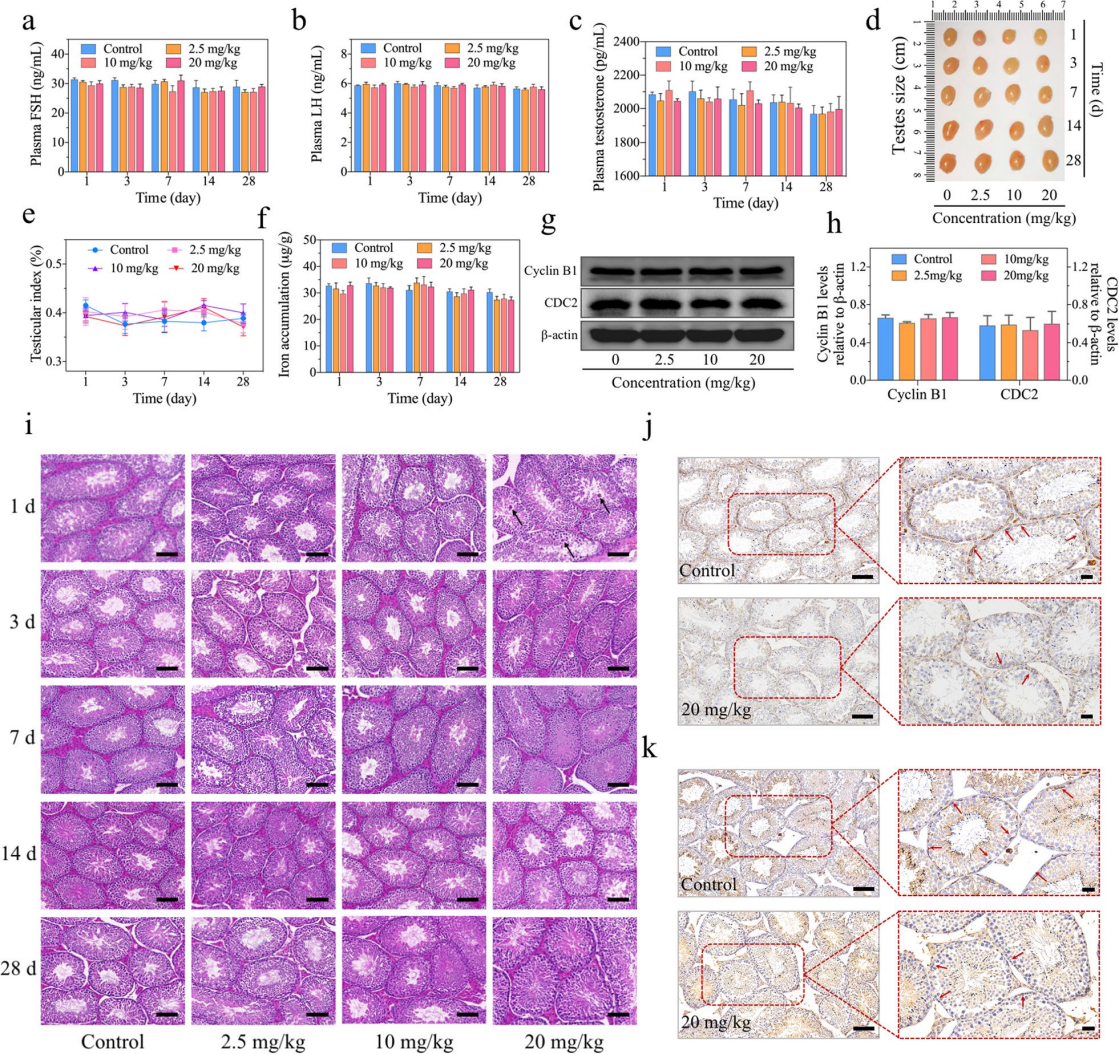
The evaluation of hormones and the effects on testes in male ICR mice with intravenous injection of IONPs. **a–c** The levels of hormones that were highly correlated with spermatogenesis in ICR mice at 1, 3, 7, 14 and 28 days after intravenous injection of IONPs, including FSH (**a**), LH (**b**) and testosterone (**c**). **d–f** The photo of testes (**d**), unilateral testicular index-changing curves (**e**) and accumulation of Fe ions in testes evaluated by ICP-AES (**f**). Data were expressed as the Mean ± S.E.M., n = 5. **g**, **h** CDC2 and Cyclin B1 protein levels detected by western blotting (**g**) and further quantified by ImageJ software (**h**) in male ICR mice at 1 day after intravenous injection of IONPs with various concentrations. Data were representative of three independent experiments, β-actin served as a loading control. **i** Histopathological examination of testes in ICR mice at 1, 3, 7, 14 and 28 days after intravenous injection of IONPs with various concentrations. Abnormal cells were indicated by black arrows. Scale bar, 100 μm. **j**,** k** Evaluation of N-cadherin (**j**) and Occludin (**k**) by immunohistological staining. Positive staining was indicated by red arrows. Scale bar, 100 μm

Sperms are produced in seminiferous tubules of the testis, followed by being transported and stored in the epididymis for leaving the male body (Fig. 4e). Therefore, the damage to testis or/and epididymis may be potential causes of the decline of sperm quantity and quality after injection with IONPs. Normal testicular function, being dependent upon hormones acting through endocrine or paracrine manners, is essential for germ cell homeostasis in vivo [49]. Among these hormones, follicle-stimulating hormone (FSH), luteinizing hormone (LH) and testosterone (T) are particularly important to male germ cell fate, as the disorder of them would induce aberrant sperm parameters and even germ cell apoptosis [50]. To explore whether the reduction of sperm quantity and quality was associated with the abnormal levels of hormones in the mice injected with IONPs, we measured FSH, LH and testosterone, respectively. The results revealed the three hormones in mice treated with IONPs fluctuated within the normal ranges (Fig. 5a–c). Specifically, the levels of FSH (29.99 ± 0.99 ng/mL) and LH (5.93 ± 0.08 ng/mL) in mice treated with 20 mg/kg IONPs at the first day were almost equivalent to the control group (31.40 ± 0.53 ng/mL and 5.87 ± 0.04 ng/mL). In addition to these two hormones that secreted by pituitary gland, testosterone also presented the negligible alternation between the high-dose IONPs-injected group (2045.09 ± 13.14 pg/mL) and the control group (2084.35 ± 14.48 pg/mL) at the first day post-injection. Therefore, the normal hormone levels indicated that the damage to the testis of IONPs was extremely limited or even absent. Later on, to further explore the cause of the sperm loss and disability, we observed the testicular size, weight and shape in each groups. The results showed that there were no significant changes in testicular size and shape among the mice injected with IONPs and the comparable control groups (Fig. 5d). Concretely, testicular weight increased from 105.80 ± 2.15 mg of the first day to 142.20 ± 2.87 mg of the 28th day after 20 mg/kg IONPs injection, which tendency was also observed in the other groups (Additional file 1: Fig. S11). To exclude the effect of age on testicular weight and more intuitively understand testicular changes, testicular index was introduced to further evaluation, which also verified the negligible differences among each groups (Fig. 5e), indicating that the macro-damage of IONPs to the testis was ignorable.

Unlike most other organs, testis possesses a unique structure called blood-testis barrier (BTB) that is composed of connective tissue, capillary endothelium, basal membrane of spermatogenic epithelium and Sertoli cell tight junction, which could not only prevent sperm antigens from escaping outside the spermatogenic tubules but also avoiding harmful substances from entering the testis to maintain environmental homeostasis for sperm development [51]. Therefore, to identify whether IONPs could enter and accumulate in the testis, ICP-AES was used to detect the abundance of Fe ions. Compared with the control group, there was no additional accumulation of Fe ions in the testis of mice injected with IONPs at each timepoints (Fig. 5f). Even at the first day after injection of IONPs that the sperm quantity and quality decreased most obviously, the concentration of Fe ions in the control group (32.75 ± 0.71 μg/g) was not significantly lower than that in the low-, middle- and high-dose groups administrated with IONPs (31.60 ± 2.20, 29.73 ± 1.20 and 32.86 ± 1.26 μg/g, respectively). These results confirmed that IONPs have not entered into the testis with detectable amounts.

### Observation of the testicular function after IONPs administration

The index to reflect the spermatogenesis is an important reference for judging testicular tissue injury, but not the only criterion. The combination of catalytic subunit cell division cycle 2 (CDC2, also known as cyclin-dependent kinase 1, CDK1) and the regulatory subunit Cyclin B1 are generally accepted to form the heterodimer complex, which are essential during spermatogenesis [52, 53]. Given that the sperm parameters altered dramatically at 1 day post-injection of IONPs (Fig. 4a–d), we have detected the protein levels of CDC2/Cyclin B1 in the testis of the male ICR mice 1 day after initial intravenous injection of IONPs with low, middle and high concentrations. As a result, there was no significant change of the CDC2/Cyclin B1 levels after IONPs exposure, compared with that in the control group (Fig. 5g, h). In addition to the detection of the overall testicular function, slight injury which may cause the subtle changes of the testis, should also be carefully assessed. Accordingly, the H&E staining was used to display the structures of the testis. As a result, the structures have not shown significant differences between the control group with low- and middle-dose IONPs injection groups, while a few scattered abnormal cells were observed in the testis of mice with high-dose injection of IONPs on the first day (Fig. 5i), suggesting high-dose IONPs might cause the limited apoptotic cell death in testis. Johnsen scoring is routinely used for histologically grading in testis H&E staining, which is an effective and quantitative method to evaluate histological features and morphological changes to various pathological factors affecting testicular cells, mostly when there is no immediate or only minimal clinical alteration [54]. In this study, we used the standard Johnsen scoring system to evaluate the pathological changes of testis. As shown in Additional file 1: Fig. S12, at the first day after injection with low- and middle-dose IONPs, the Johnsen scores of the testis were both 9.67 ± 0.21, almost the same with that of the control group, while after the high-dose IONPs injection (20 mg/kg), the Johnsen score of the testis (9.17 ± 0.31) was significantly lower than the control group (9.83 ± 0.17), suggesting slightly impaired spermatogenesis, which was characterized by increased late spermatids and disorganized epithelium in testis [54]. Consistent with the observation of sperm parameters and other indicators, the most severe damage occurred at the first day after IONPs injection and gradually recovered within 2 weeks (Additional file 1: Fig. S12). However, as the presence of BTB prevented the entry of IONPs into the testis, it was quite interesting to explore why did the apoptotic cells appear after high-dose IONPs injection. In testis, there is a population of cells named Sertoli cell, which has receptors for the hormonal regulators of spermatogenesis such as FSH and testosterone, and also provides paracrine factors, nutrients, cytokines and biologically active peptides that essential for supporting spermatogenesis and fertility [55]. Notably, BTB is created by adjacent Sertoli cells near the basement membrane to divide the seminiferous epithelium into the basal and adluminal (apical) compartments, and subsequently serves for successful postmeiotic spermatid development in the apical compartment [56]. Sertoli cells on the front lines might be damaged by those toxic substances and lead to functional impairment or even cell death in the testis. As no morphological abnormalities in the Sertoli cells were observed from H&E staining of testis, we hypothesized that the extremely low-amount IONPs entered into the Sertoli cells and impaired their function, consequently leading to the apoptosis of a few spermatogonia. To verify this hypothesis, we have detected the markers near the basal membrane to evaluate the function of Sertoli cells. Among these markers, Occludin is a tight junction protein, which participates in forming the paracellular barrier that mediates the communications of substances in the intercellular compartments between Sertoli cells [57], and N-cadherin is responsible for cell adhesion and recognition [58]. The immunohistochemical (IHC) images revealed that IONPs injection decreased the local expression and density of the markers (Fig. 5j, k). Among them, the tight junction supported by Occludin became discontinuous, which further indicated that the function of the Sertoli cells was partially impaired (Fig. 5k). Collectively, Sertoli cells may internalize a tiny amount of IONPs when preventing them from entering the testis, which decreased their own ability to nourish the spermatogonia and resulted in a few apoptotic spermatogonia. Notably, although low- and middle-dose administration of IONPs in mice elicited significant sperm quantity and quality decline within seven days (Fig. 4a–d), extremely limited or no impairment of Sertoli cells have been observed in those mice, suggesting the abnormal sperm parameters elicited by IONPs, to a large extent, were not attributed to the limited Sertoli cells impairment.

### Investigation of the toxicity of IONPs to epididymis in vivo

**Fig. 6:**
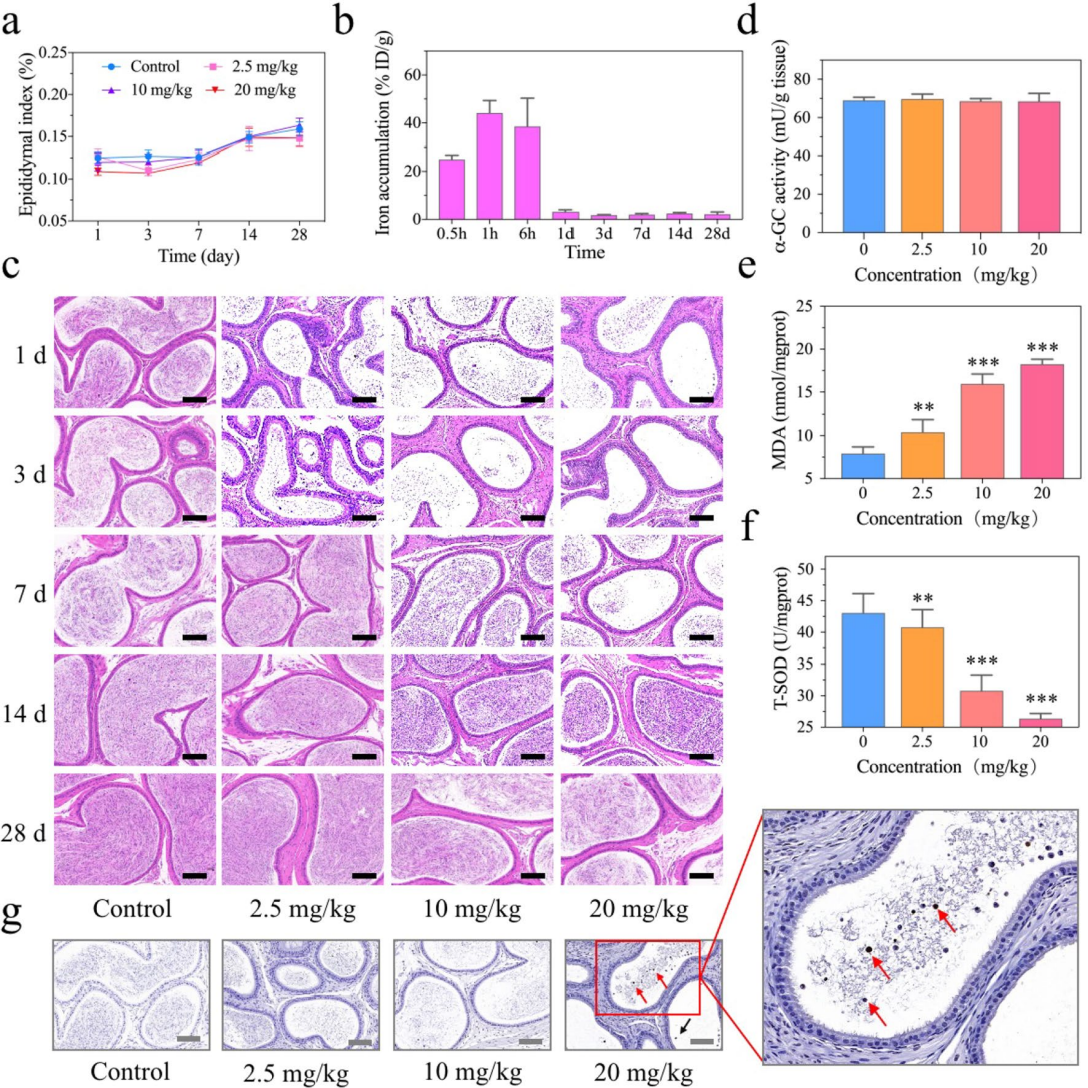
The effects of IONPs on epididymides in male ICR mice. **a–c** Unilateral epididymal index-changing curves (**a**), accumulation of Fe ions in epididymides evaluated by ICP-AES (**b**) and histopathological examination of epididymides (**c**) in ICR mice at 1, 3, 7, 14 and 28 days after intravenous injection of IONPs with various concentrations. Scale bar, 100 μm. **d–f** The α-GC activity (**d**) and indexes of oxidative stress in epididymides of ICR mice at 1 day after intravenous injection of IONPs, including MDA (**e**) and T-SOD (**f**). Data were expressed as the Mean ± S.E.M., n = 5, **P < 0.01, ***P < 0.001. **g** TUNEL staining of epididymides from sacrificed ICR mice at 1 day after low-, middle- and high-dose IONPs injection. Apoptotic cells were indicated by arrows. Scale bar, 100 μm

As mentioned above, the damage to testis or/and epididymis may be potential causes of the abnormal sperm parameters in mice injected with IONPs. Since testis has not been influenced by IONPs obviously, it makes sense to explore whether epididymis has been impaired after IONPs injection and subsequently caused sperm quantity and quality reduction. Immature sperms from the testis will be transported into the epididymis to form mature sperms followed by storage during asexual activity. Thus, the significant reduction of sperms in epididymis of mice injected with IONPs at the first day was probably not correlated with the decreased production of immature sperms or the presence of very few apoptotic cells in the testis. In contrast, we speculated that the reduction of sperm counts in epididymis might be attributed to the direct toxicity of IONPs, as the deterioration of the epididymal environment could also lead to the death of mature sperms. Herein, epididymis index was firstly measured to evaluate epididymal changes. At the first day after injection of IONPs, there were no statistical differences of epididymis index between the control group (mean value, 0.12%) and even the high-dose IONPs injection group (mean value, 0.10%) (*P* > 0.05). With the growth of mice, on the 28th day after injection, the epididymis index in all mice increased, but there were still no statistical differences between the control group with each IONPs-injected groups (*P* > 0.05) (Fig. 6a). In addition, we have assessed the weight change of epididymis and recorded the remarkably similar values between the control group with each administration groups (*P* > 0.05) (Additional file 1: Fig. S13). Moreover, due to the absence of BTB, we speculated that IONPs could directly enter the epididymis and subsequently accumulated, leading to the death of sperms. This hypothesis was confirmed via the ICP-AES analysis, demonstrated by that the concentration of Fe ions rapidly accumulated in the epididymis to a maximum value of 44.15 ± 5.20% ID/g after 1 h, while rapidly dropping to an extremely low level within 24 h (1.99 ± 0.35% ID/g) (Fig. 6b). The tendency coincided with the decline and recovery time of sperm counts and suggested that the decrease of sperms in the epididymis was tightly correlated with the accumulation of IONPs, characterized by Fe ions in ICP-AES analysis. In addition to the evaluation of the quantity and quality by analyzing sperms in the suspension, H&E staining was also applied to directly observe sperms in the epididymis. The presence of viable sperms in the epididymal lumen was hardly observed at the first day after injection with IONPs. As time increased, the number of sperms continued to increase until it returned to the normal range at the 14th day after administration (Fig. 6c). These results were consistent with the parameters revealed by a computer-assisted semen analysis system (Fig. 4a–d). To explore the underlying mechanism regarding the reversible sperm abnormality, we have firstly detected the α-glucosidase (α-GC) activity in the epididymis of the male ICR mice 1 day after intravenous injection of IONPs with low, middle and high concentrations, as α-GC enzyme activity can be used as a sensitive indicator of epididymal function [59]. As a result, the α-GC enzyme activities were 69.67 ± 2.61, 68.53 ± 1.36, 68.49 ± 4.16 mU/g tissue after low-, middle- and high-dose IONPs injection, respectively, and there were no significant difference compared with the value of the control group (68.97 ± 1.66 mU/g tissue), suggesting the normal epididymal function (Fig. 6d). Notably, previous studies have shown that the accumulation of intracellular Fe ions could augment the Fenton reaction to produce a large number of harmful oxidative products [60]. Therefore, oxidative stress may be the underlying mechanism of sperm death. Malondialdehyde (MDA) is well-known as one of the final products of polyunsaturated fatty acids peroxidation and the increased oxidative stress would cause the accumulation of MDA [61]. However, superoxide dismutase (SOD) is commonly regarded as a natural scavenger of free radicals and main antioxidant enzyme to fight against oxidative stress in the body [62]. To assess the extent of oxidative stress in epididymis, the levels of MDA and the activities of SOD were measured (Fig. 6e, f). In the group injected with high-dose (20 mg/kg) IONPs, the MDA level was 18.26 ± 0.57 nmol/mgprot, which was significantly higher than the value of the control group (7.93 ± 0.78 nmol/mgprot) (Fig. 6e). Meanwhile, the SOD activity of 43.05 ± 3.10 U/mgprot in the control group decreased to 26.37 ± 0.82 U/mgprot in the high-dose IONPs injection group (Fig. 6f). These results together suggested that IONPs elevated the oxidative stress in the epididymis. Since oxidative stress is a potent stimulator of apoptosis [63], to further evaluate the underlying mechanism regarding the reversible sperm quantity and quality decline after IONPs injection, TUNEL staining was firstly applied to evaluate the apoptotic activity. As shown in Fig. 6g, apoptotic cells were observed in the epididymal lumen especially after the high-dose IONPs administration, the shape and size of which were similar with the abnormal cells indicated by H&E staining of testis, suggesting the partial apoptotic cells observed by TUNEL staining may originate from the slightly impaired testis. Furthermore, as caspase-3 is at the end of the caspase cascade and can be activated by both the intrinsic and extrinsic death pathways, the best-known apoptotic executor/marker cleaved caspase-3 has been utilized to mark and monitor the apoptotic sperms in the epididymis [64, 65]. As a result, the positive-staining pattern of cleaved caspase-3 via IHC staining was in accordance with the reversible sperm quantity and quality decline revealed by H&E staining and computer-aided semen analysis (Additional file 1: Fig. S14).

Collectively, we have identified that the elevated oxidative stress levels and consequently induced apoptosis caused by the accumulation of IONPs in the epididymis might be one of the underlying mechanisms for the rapid decline of sperm parameters. Based on the consistently altered pattern of the apoptotic activity and sperm parameters, we speculated that the recovery of the sperm quantity and quality might be attributed to the re-established oxidative stress balance and decreased apoptotic activity in epididymis. Given that the sperm damage was temporary and reversible, it may provide some important implications in clinical practice. It is well established that the spermatogenic cycle is critical for continuous sperm production, and the spermatogenic cycle of mice is eight weeks [66]. In consideration of that the reduction of sperm quantity and quality caused by the IONPs injection was able to completely return to the normal range within two weeks, we speculated that the sperm parameters would be free of abnormalities within a quarter of spermatogenic cycle in male patients who have received MRI examination with IONPs injection, in other words, the fertilized behavior of them was strongly recommended after about 2.5 weeks, which is a quarter of spermatogenic cycle of human beings (10 weeks) [67].

## Conclusions

In summary, we have revealed a unique and temporary side effect of IONPs as *T*_2_-MRI contrast agents in vivo. Although the systemic toxicities to major organs were negligible after IONPs administration, the male reproductive toxicity should be concerned. The Sertoli cells damage caused by IONPs led to pathological changes and even limited apoptosis of spermatogenic cells in the testis. More importantly, the rapid accumulation of IONPs exerted considerable cytotoxicities to the sperms in the storage organ epididymides, which might be associated with the elevated oxidative stress and apoptosis. However, the impairment elicited by IONPs was temporary and reversible, by returning to the normal range within 14 days, indicating that the male patients who have received MRI examination with IONPs as contrast agents should carefully delay the fertilized behavior after a quarter of one spermatogenic cycle period. Collectively, we believe these findings may provide new insights in clinical practice and for better avoiding the side effects that were easy to be negligible with contrast agents administration.

## Supplementary Information

**Additional file 1: Figure S1**. TEM image of monodisperse iron oxide nanocrystals (IONCs). **Figure S2**. The FT-IR spectra of oleic acid-capped ION and IONPs. **Figure S3**. EDS spectrum of IONPs. **Figure S4**. (a) XPS spectrum of IONPs recorded in the range of 0-1300 eV. (b) Fe 2p XPS spectrum of the IONP. The two peaks at 711 eV and 725 eV in Fe 2p spectrum indicated the magnetite phase. (c) O 1s and (d) C 1s XPS spectra. **Figure S5**. Thermogravimetric curve of IONPs under N_2_ at a rate of 10 °C/min. **Figure S6**. The absorbance (OD 545 nm) of the supernatant of red blood cell suspension incubated with IONPs for 4 h. **Figure S7**. Histopathological examination of the major organs of ICR mice after intravenous injection of IONPs at various concentrations for 3 days. **Figure S8**. Histopathological examination of the major organs of ICR mice after intravenous injection of IONPs at various concentrations for 7 days. **Figure S9**. Histopathological examination of the major organs of ICR mice after intravenous injection of IONPs at various concentrations for 14 days. **Figure S10**. Histopathological examination of the major organs of ICR mice after intravenous injection of IONPs at various concentrations for 28 days. **Figure S11**. The changes of testicular weight of of ICR mice after intravenous injection of IONPs. **Figure S12**. Johnsen scores of testis of ICR mice after intravenous injection of IONPs. **Figure S13**. The changes of epididymal weight of of ICR mice after intravenous injection of IONPs. **Figure S14**. Immunohistochemical staining of cleaved caspase-3 in the epididymis of male ICR mice at 1, 3, 7, 14 and 28 days after intravenous injection of IONPs with various concentrations.

## Data Availability

Most of the datasets supporting the conclusions of this study are included within the manuscript and the additional files. The datasets used or analyzed during the study are available on reasonable request.

## Abbreviations

IONPs: Iron oxide nanoparticles
MRI: Magnetic resonance imaging
RES: Reticuloendothelial system
BTB: Blood testicular barrier
CT: Computerized tomography
*T*_1_/*T*_2_-WI: *T*_1_/*T*_2_-Weighted imaging
DWI: Diffusion-weighted imaging
DCEI: Dynamic contrast-enhanced imaging
SPIONs: Superparamagnetic iron oxide nanoparticles
FDA: Food and drug administration
ESIONs: Extremely small iron oxide nanoparticles
MnO NPs: Manganese oxide nanoparticles
GBCAs: Gadolinium-based contrast agents
IONCs: Iron oxide nanocrystals
DMSA: Dimercaptosuccinic acid
ROS: Reactive oxygen species
MDA: Malondialdehyde
ZnO NPs: Zinc oxide nanoparticles
PAA: Poly (acrylic acid)
ALT: Alanine aminotransferase
AST: Aspartate aminotransferase
Cr: Creatinine
BUN: Blood urea nitrogen
DMSO: Dimethyl sulfoxide
ICP-AES: Inductive coupled plasma atomic emission spectrometer
T-SOD: Total superoxide dismutase
SEM: Standard error of mean
