# Supplementary material for: Effects of iron oxide nanoparticles as T2-MRI contrast agents on reproductive system in male mice
Source: J Nanobiotechnology. 2022 Mar 2;20:98. doi: 10.1186/s12951-022-01291-2 (PMC8889634; doi:10.1186/s12951-022-01291-2)
Supplement: Supplementary file 1 — Additional file 1: Figure S1. TEM image of monodisperse iron oxide nanocrystals (IONCs). Figure S2. The FT-IR spectra of oleic acid-capped ION and IONPs. Figure S3. EDS spectrum of IONPs. Figure S4. (a) XPS spectrum of IONPs recorded in the range of 0-1300 eV. (b) Fe 2p XPS spectrum of the IONP. The two peaks at 711 eV and 725 eV in Fe 2p spectrum indicated the magnetite phase. (c) O 1s and (d) C 1s XPS spectra. Figure S5. Thermogravimetric curve of IONPs under N2 at a rate of 10 °C/min. Figure S6. The absorbance (OD 545 nm) of the supernatant of red blood cell suspension incubated with IONPs for 4 h. Figure S7. Histopathological examination of the major organs of ICR mice after intravenous injection of IONPs at various concentrations for 3 days. Figure S8. Histopathological examination of the major organs of ICR mice after intravenous injection of IONPs at various concentrations for 7 days. Figure S9. Histopathological examination of the major organs of ICR mice after intravenous injection of IONPs at various concentrations for 14 days. Figure S10. Histopathological examination of the major organs of ICR mice after intravenous injection of IONPs at various concentrations for 28 days. Figure S11. The changes of testicular weight of of ICR mice after intravenous injection of IONPs. Figure S12. Johnsen scores of testis of ICR mice after intravenous injection of IONPs. Figure S13. The changes of epididymal weight of of ICR mice after intravenous injection of IONPs. Figure S14. Immunohistochemical staining of cleaved caspase-3 in the epididymis of male ICR mice at 1, 3, 7, 14 and 28 days after intravenous injection of IONPs with various concentrations. [file 12951_2022_1291_MOESM1_ESM.docx]

**Supporting Information**

**Effects of iron oxide nanoparticles as *T*_2_-MRI contrast agents on reproductive system in male mice**

Heyu Yang^1†^, Hui Wang^1†^, Chenghao Wen^1^, Shun Bai^2^, Pengfei Wei^3^, Bo Xu^2^, Yunjun Xu^2^, Chaozhao Liang^1^, Yunjiao Zhang^4^, Guilong Zhang^3*^, Huiqin Wen^5*^ and Li Zhang^1,6,7,*^

^*^Correspondence:

[glzhang@bzmc.edu.cn](mailto:glzhang@bzmc.edu.cn); [wenhuiqin@ahmu.edu.cn](mailto:wenhuiqin@ahmu.edu.cn); [lzhang@ahmu.edu.cn](mailto:lzhang@ahmu.edu.cn)

^†^ Heyu Yang and Hui Wang contributed equally to this work

^1^ Department of Urology, the First Affiliated Hospital of Anhui Medical University, Institute of Urology, Anhui Medical University and Anhui Province Key Laboratory of Genitourinary Diseases, Anhui Medical University, Hefei 230022, China.

^2^ Reproductive and Genetic Hospital, Department of Radiology, Anhui Provincial Hospital, the First Affiliated Hospital of USTC, Division of Life Sciences and Medicine, University of Science and Technology of China, Hefei 230001, China.

^3^ School of Pharmacy, the Key Laboratory of Prescription Effect and Clinical Evaluation of State Administration of Traditional Chinese Medicine of China, Binzhou Medical University, Yantai 264003, China.

^4^ School of Medicine and Institutes for Life Sciences, South China University of Technology, Guangzhou 510006, China.

^5^ Department of Blood Transfusion, the First Affiliated Hospital of Anhui Medical University, Hefei 230022, China.

^6^ Center for Scientific Research of the First Affiliated Hospital of Anhui Medical University, Hefei 230022, China.

^7^ Anhui Provincial Institute of Translational Medicine, Hefei 230032, China.

Full list of author information is available at the end of the article


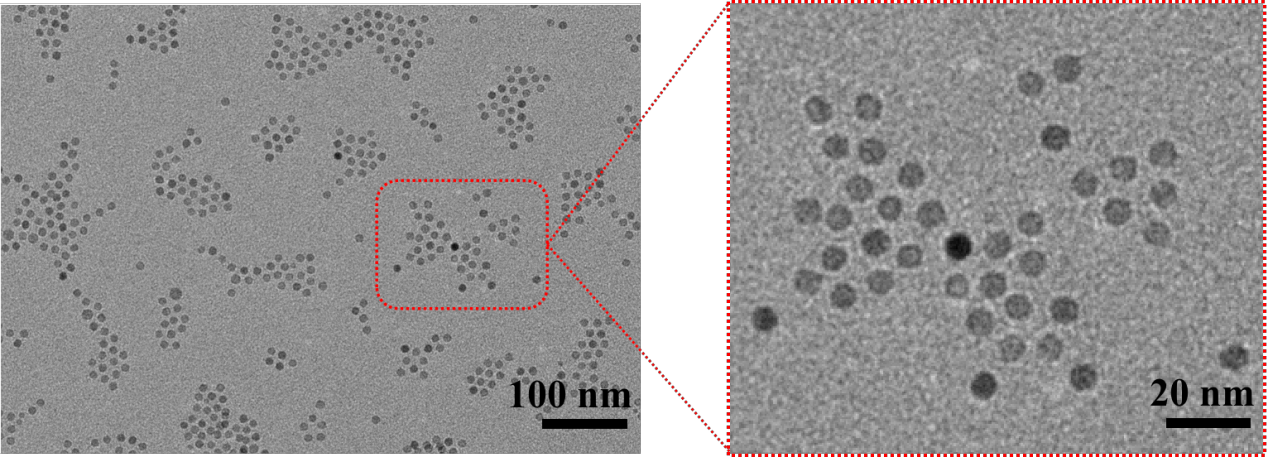


**Fig. S1** TEM image of monodisperse iron oxide nanocrystals (IONCs).


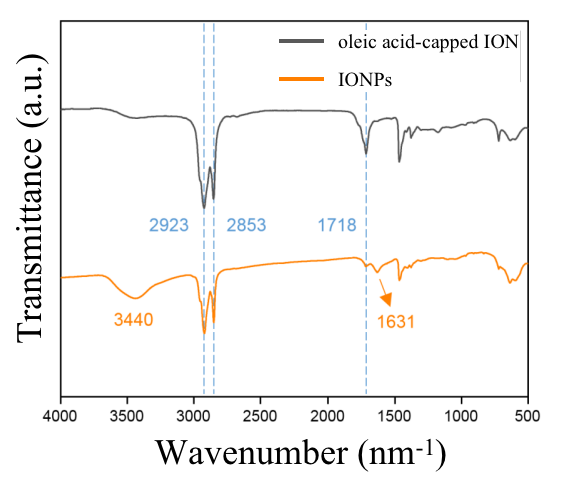


**Fig. S2** The FT-IR spectra of oleic acid-capped ION and IONPs.


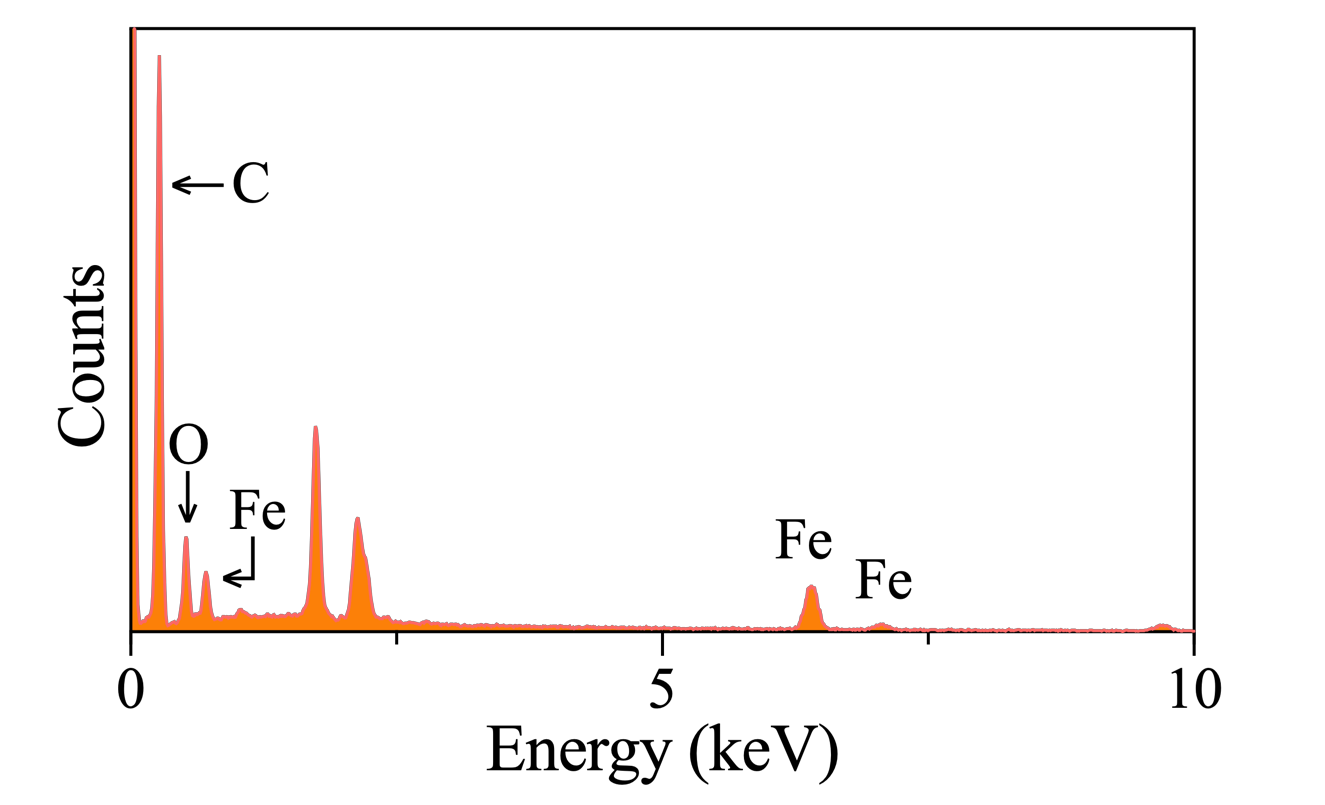


**Fig. S3** EDS spectrum of IONPs.


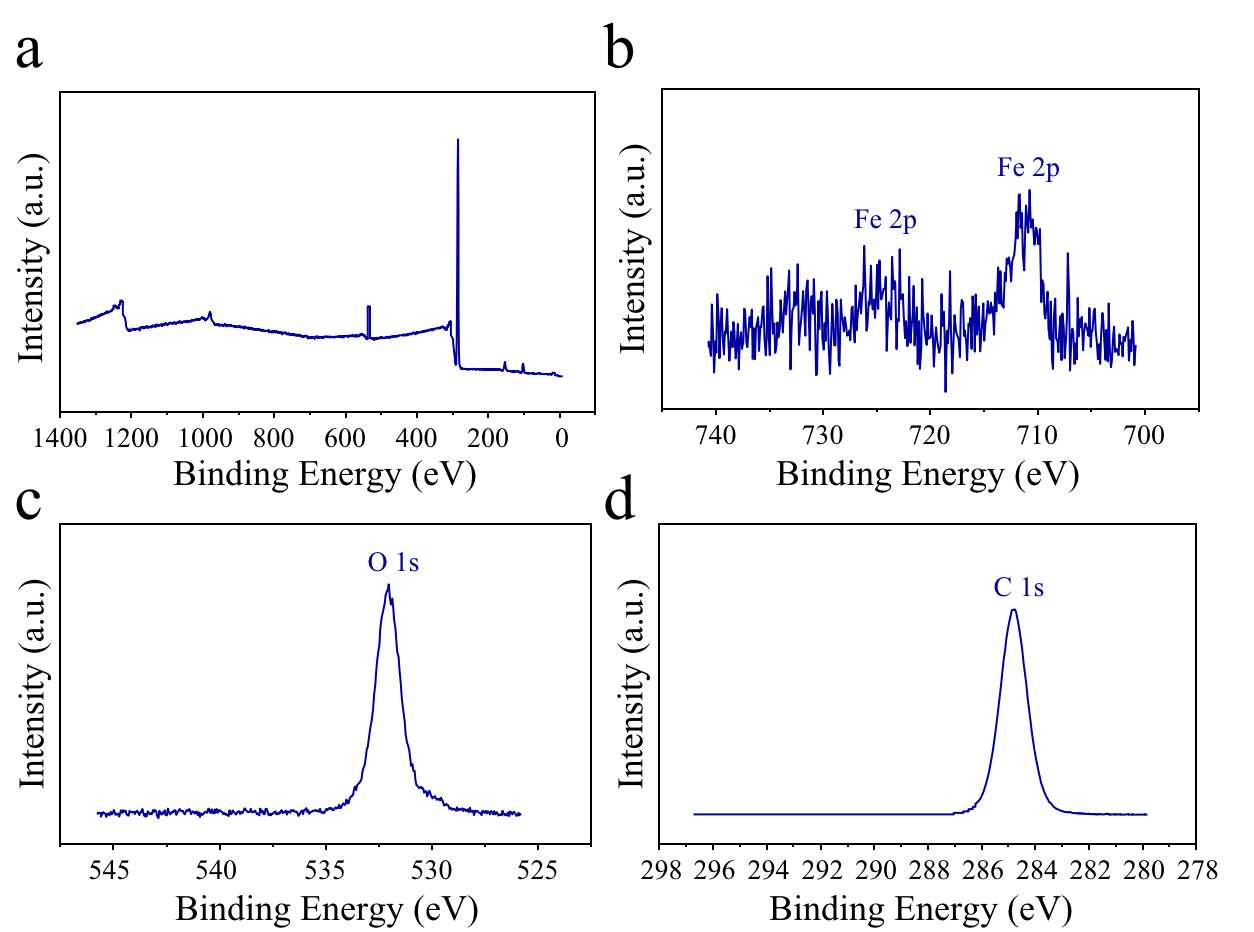


**Fig. S4** (**a**) XPS spectrum of IONPs recorded in the range of 0-1300 eV. (**b**) Fe 2p XPS spectrum of the IONP. The two peaks at 711 eV and 725 eV in Fe 2p spectrum indicated the magnetite phase. (**c**) O 1s and (**d**) C 1s XPS spectra.


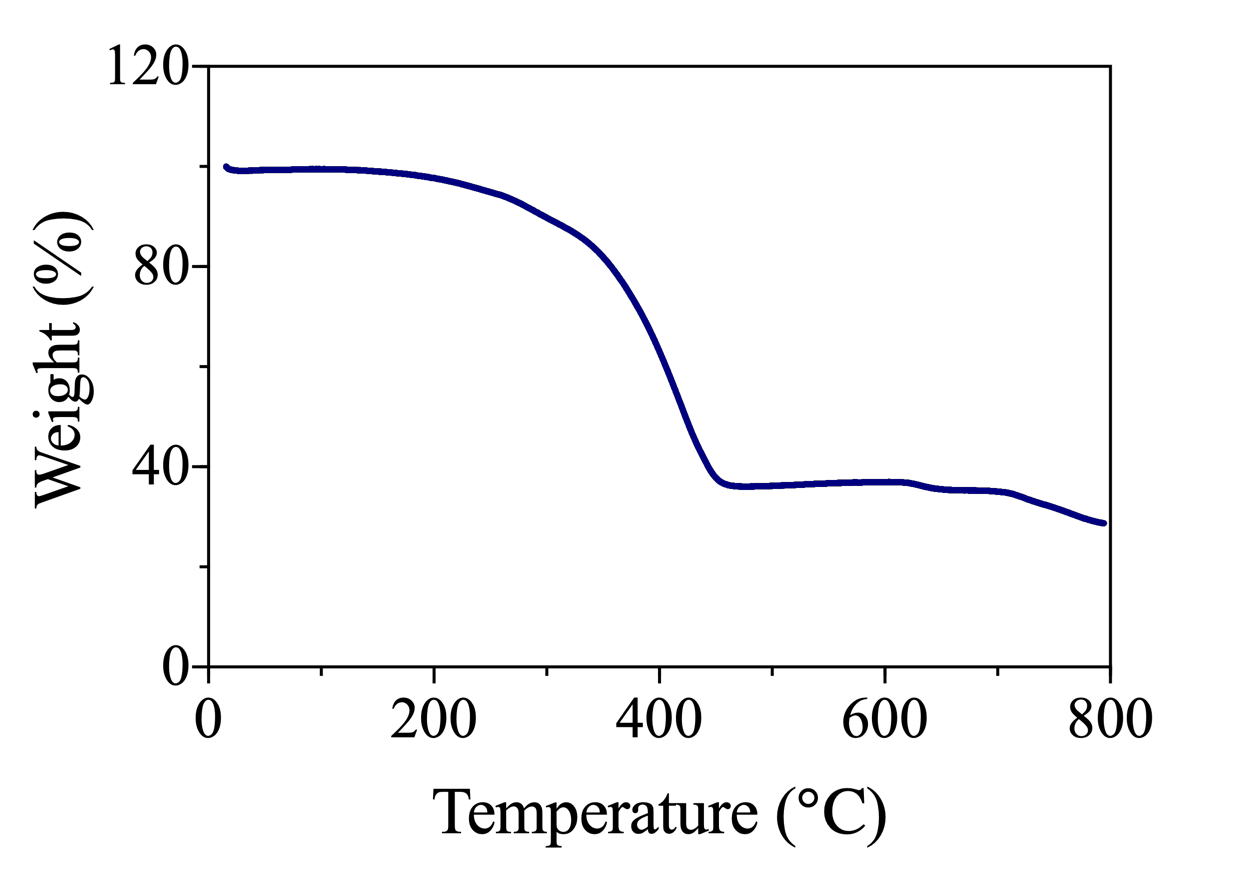


**Fig. S5** Thermogravimetric curve of IONPs under N_2_ at a rate of 10 °C/min.


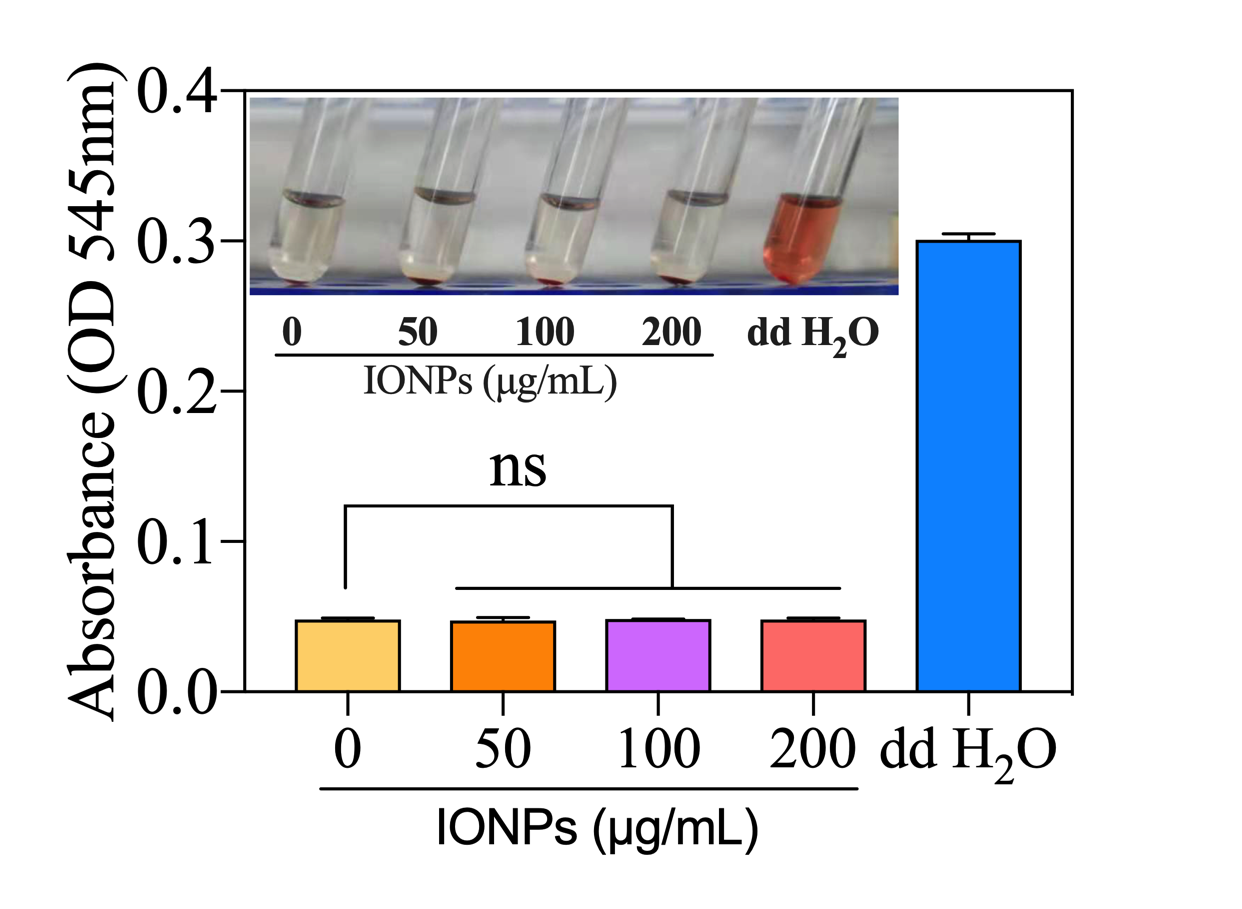


**Fig. S6** The absorbance (OD 545 nm) of the supernatant of red blood cell suspension incubated with IONPs for 4 h. Data were expressed as the Mean ± S.E.M., n=3, ns, not significant.


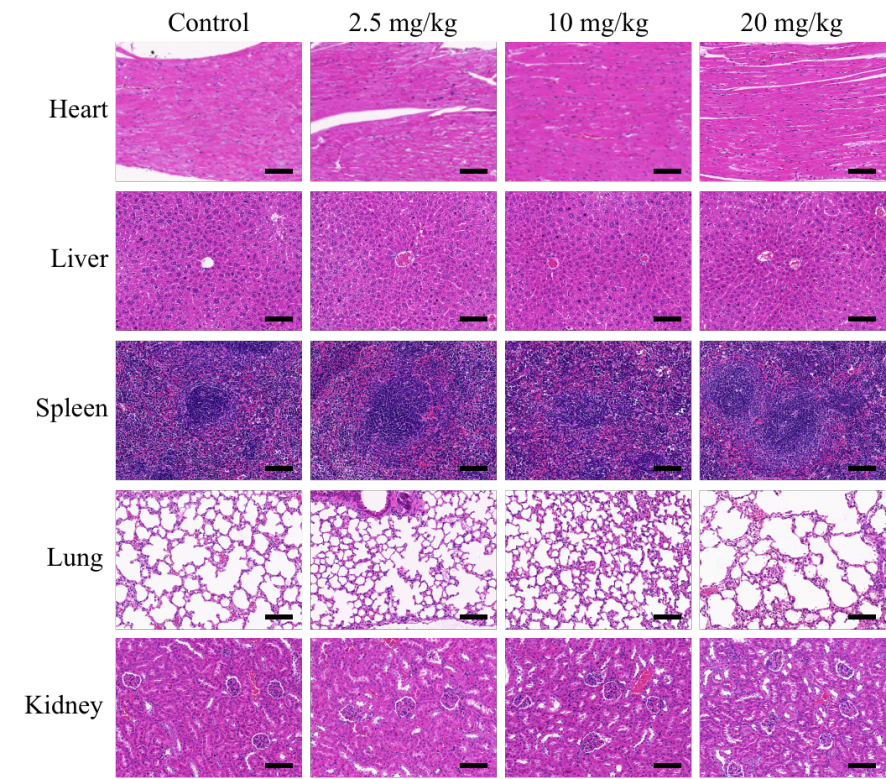


**Fig. S7** Histopathological examination of the major organs of ICR mice after intravenous injection of IONPs with various concentrations for 3 days. Scale bar, 100 μm.


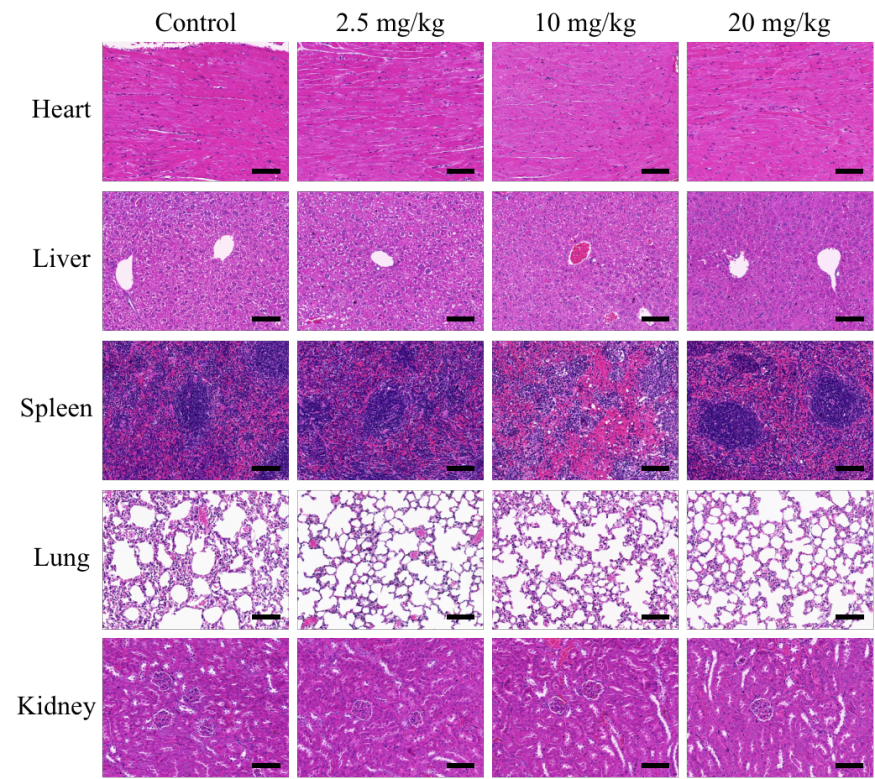


**Fig. S8** Histopathological examination of the major organs of ICR mice after intravenous injection of IONPs with various concentrations for 7 days. Scale bar, 100 μm.


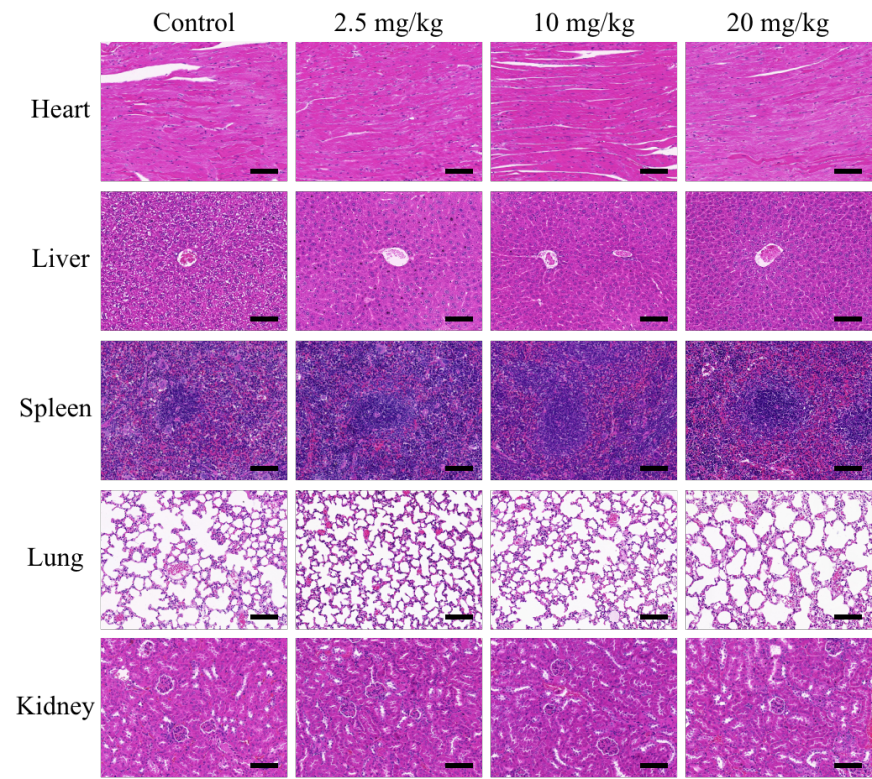


**Fig. S9** Histopathological examination of the major organs of ICR mice after intravenous injection of IONPs with various concentrations for 14 days. Scale bar, 100 μm.


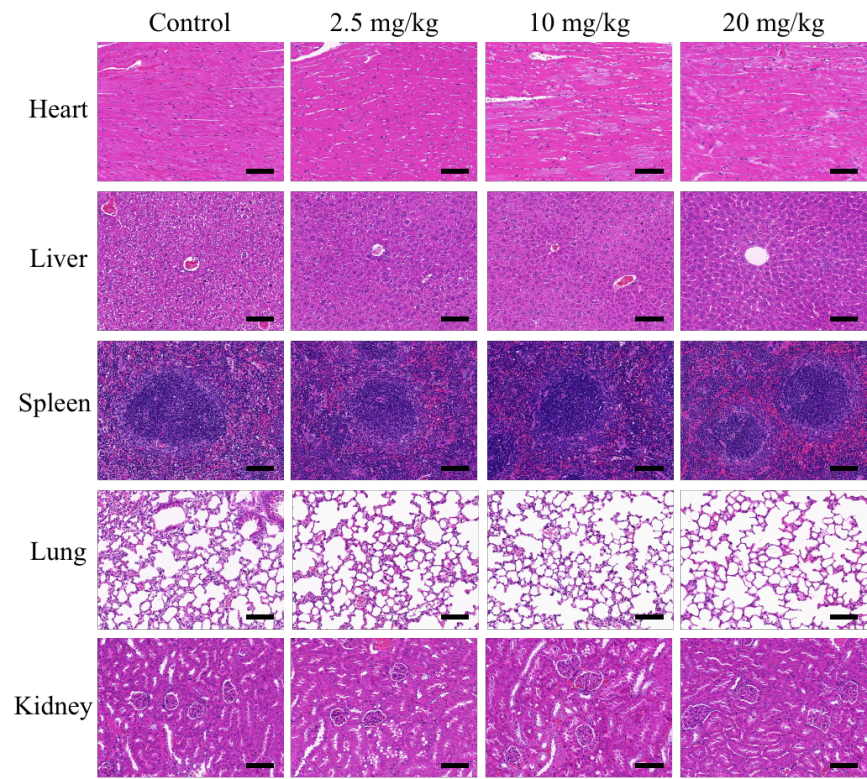


**Fig. S10** Histopathological examination of the major organs of ICR mice after intravenous injection of IONPs with various concentrations for 28 days. Scale bar, 100 μm.


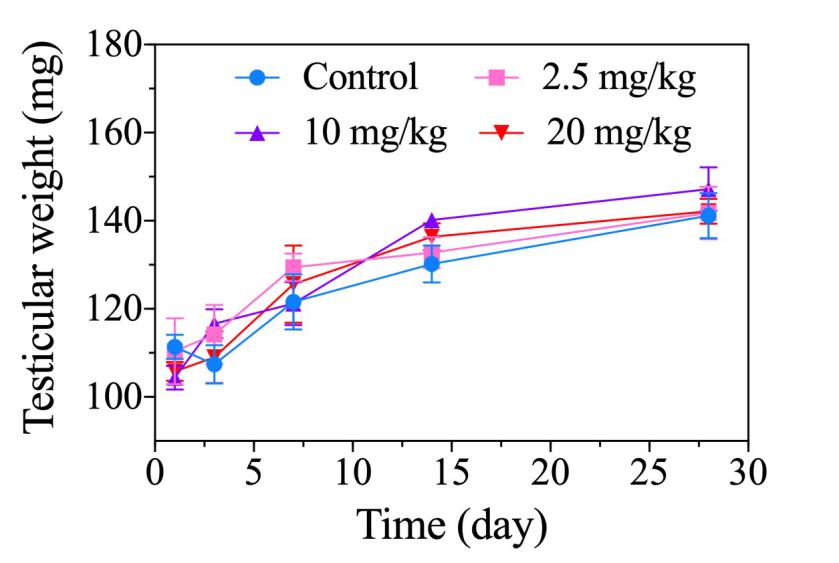


**Fig. S11** The changes of unilateral testicular weight of of ICR mice after intravenous injection of IONPs. Data were expressed as the Mean ± S.E.M., n=5.


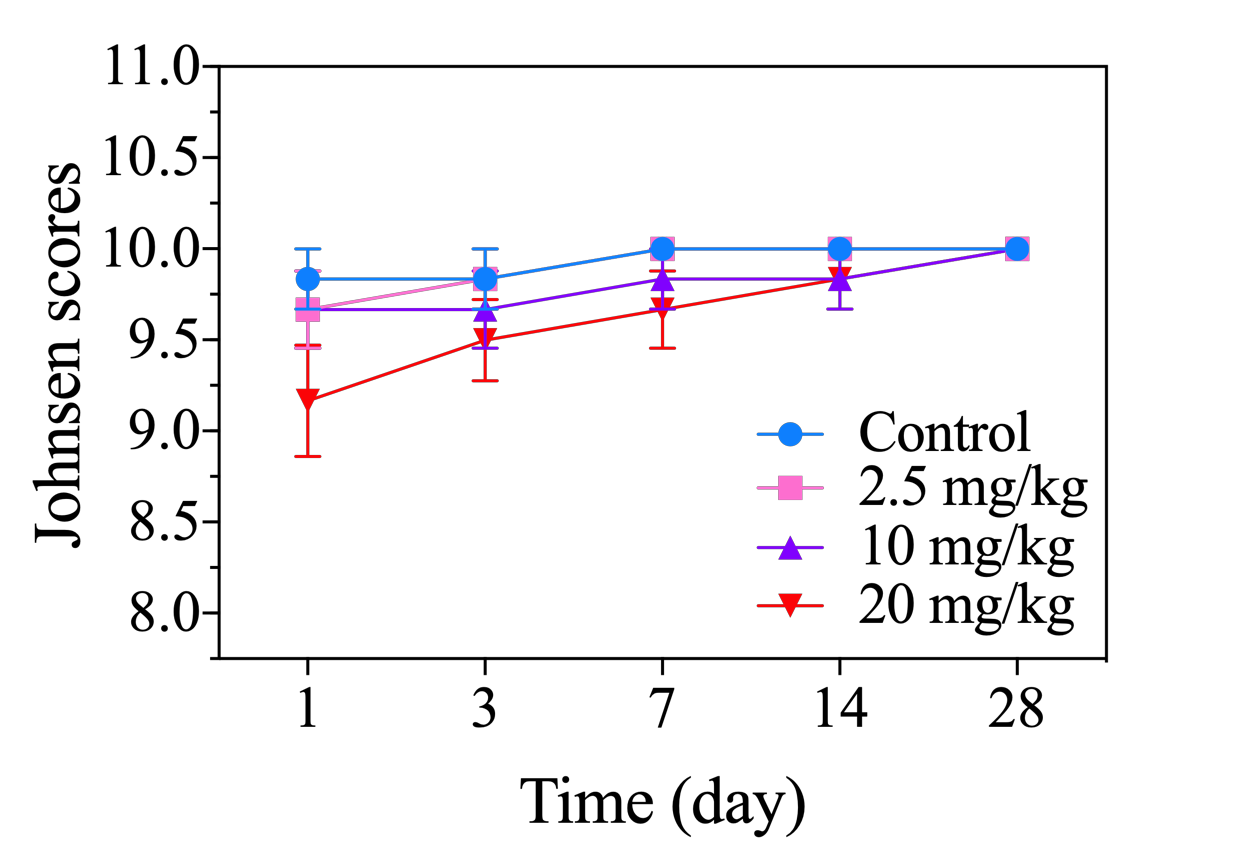


**Fig. S12** Johnsen scores of testis of ICR mice after intravenous injection of IONPs. Data were expressed as the Mean ± S.E.M., n=5.


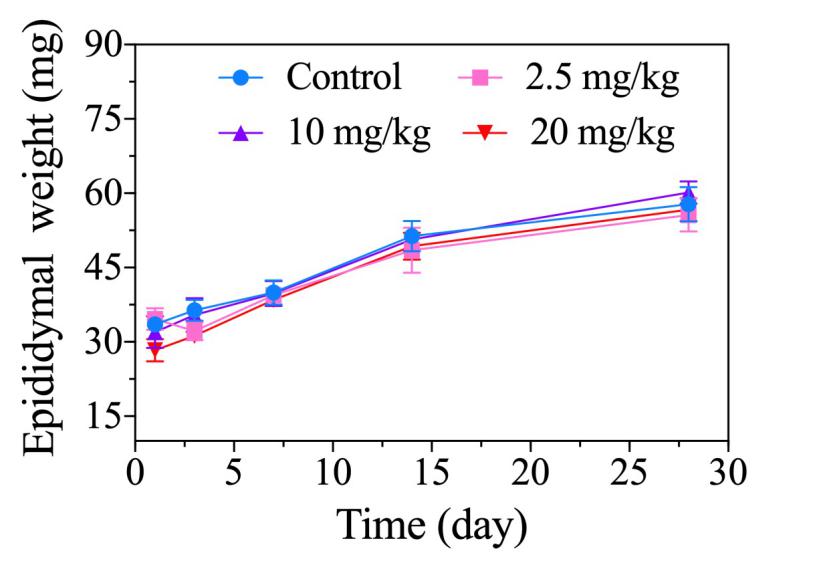


**Fig. S13** The changes of unilateral epididymal weight of of ICR mice after intravenous injection of IONPs. Data were expressed as the Mean ± S.E.M., n=5.

**
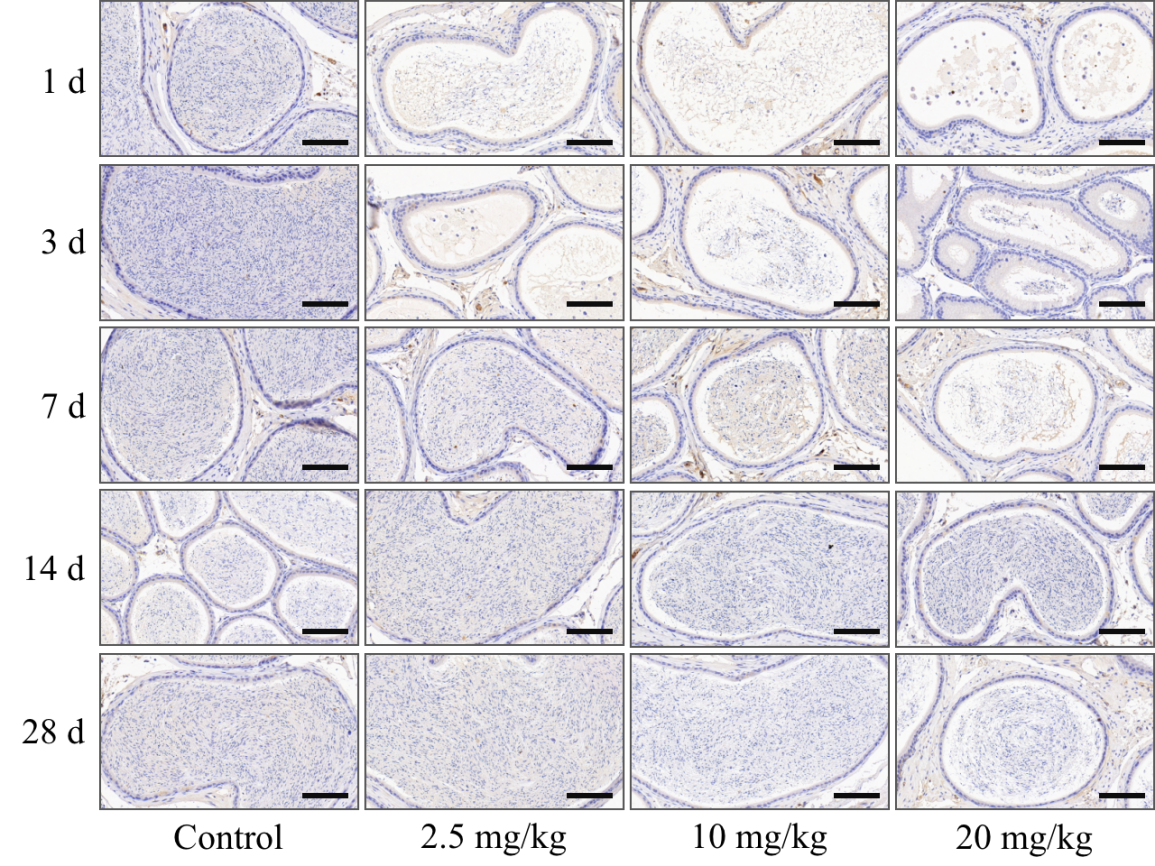
**

**Fig. S14** Immunohistochemical staining of cleaved caspase-3 in the epididymis of male ICR mice at 1, 3, 7, 14 and 28 days after intravenous injection of IONPs with various concentrations. Scale bar, 100 μm.
